# Cryogenic electron microscopy and tomography reveal imperfect icosahedral symmetry in alphaviruses

**DOI:** 10.1093/pnasnexus/pgae102

**Published:** 2024-03-07

**Authors:** David Chmielewski, Guan-Chin Su, Jason T Kaelber, Grigore D Pintilie, Muyuan Chen, Jing Jin, Albert J Auguste, Wah Chiu

**Affiliations:** Biophysics Graduate Program, Stanford University, Stanford, CA 94305, USA; Department of Bioengineering, Stanford University, Stanford, CA 94305, USA; Department of Microbiology and Immunology, Stanford University, Stanford, CA 94305, USA; Institute for Quantitative Biomedicine, Rutgers, The State University of New Jersey, Piscataway, NJ 08854, USA; Department of Bioengineering, Stanford University, Stanford, CA 94305, USA; Department of Microbiology and Immunology, Stanford University, Stanford, CA 94305, USA; Division of CryoEM and Bioimaging, SSRL, SLAC National Accelerator Laboratory, Stanford University, Menlo Park, CA 94025, USA; Vitalant Research Institute, San Francisco, CA 94118, USA; Department of Laboratory Medicine, University of California, San Francisco, CA 94143, USA; Department of Entomology, College of Agriculture and Life Sciences, Fralin Life Science Institute, Virginia Polytechnic Institute and State University, Blacksburg, VA 24061, USA; Center for Emerging, Zoonotic, and Arthropod-borne Pathogens, Virginia Polytechnic Institute and State University, Blacksburg, VA 24061, USA; Biophysics Graduate Program, Stanford University, Stanford, CA 94305, USA; Department of Bioengineering, Stanford University, Stanford, CA 94305, USA; Department of Microbiology and Immunology, Stanford University, Stanford, CA 94305, USA; Division of CryoEM and Bioimaging, SSRL, SLAC National Accelerator Laboratory, Stanford University, Menlo Park, CA 94025, USA

**Keywords:** cryo-EM, cryo-ET, alphavirus, imperfect icosahedral symmetry

## Abstract

Alphaviruses are spherical, enveloped RNA viruses with two-layered icosahedral architecture. The structures of many alphaviruses have been studied using cryogenic electron microscopy (cryo-EM) reconstructions, which impose icosahedral symmetry on the viral particles. Using cryogenic electron tomography (cryo-ET), we revealed a polarized symmetry defect in the icosahedral lattice of Chikungunya virus (CHIKV) in situ, similar to the late budding particles, suggesting the inherent imperfect symmetry originates from the final pinch-off of assembled virions. We further demonstrated this imperfect symmetry is also present in in vitro purified CHIKV and Mayaro virus, another arthritogenic alphavirus. We employed a subparticle-based single-particle analysis protocol to circumvent the icosahedral imperfection and boosted the resolution of the structure of the CHIKV to ∼3 Å resolution, which revealed detailed molecular interactions between glycoprotein E1–E2 heterodimers in the transmembrane region and multiple lipid-like pocket factors located in a highly conserved hydrophobic pocket. This complementary use of in situ cryo-ET and single-particle cryo-EM approaches provides a more precise structural description of near-icosahedral viruses and valuable insights to guide the development of structure-based antiviral therapies against alphaviruses.

**Significance Statement**

This study demonstrates the use of cryogenic electron microscopy and tomography to characterize imperfect icosahedral symmetry in alphaviruses attributable to their assembly and budding process in situ. Our advanced image processing protocol allows us to reveal previously uncharacterized interactions within the transmembrane domain of the heterodimer glycoproteins and with the capsid protein and to discover two lipid pocket factors in Chikungunya virus. Our structural discoveries can inform the development of antiviral therapies, targeting specific regions within the glycoprotein structure shared among alphaviruses.

## Introduction

The *Alphavirus* genus (family Togaviridae) contains arthropod-transmitted pathogens responsible for near-global epidemics in humans and livestock (1, 2). Chikungunya virus (CHIKV), one member of the *Alphavirus* genus, was added to the WHO shortlist for priority research, which also included coronaviruses, due to its pandemic potential (3). In 2023 and as of 31 October, ∼440,000 CHIKV cases and over 350 deaths have been reported worldwide according to the European Center for Disease Prevention and Control. CHIKV infection causes acute febrile illness accompanied by severe polyarthralgia and myalgia that can become chronic in 40–80% infected individuals (3). Global warming increases distribution of vectors for CHIKV transmission and the debilitating disease caused by CHIKV infection continues to pose threats to the global human health and economy (3). Currently, there are no licensed vaccines or treatments for CHIKV infection. Structures of alphavirus virions, including CHIKV, have been extensively studied following purification and single-particle cryogenic electron microscopy (cryo-EM) analysis. The ∼70 nm virions are composed of two icosahedral layers with an inner nucleocapsid made up with capsid proteins (Cps) and outer membrane-embedded glycoprotein shell formed by envelope glycoproteins (E1–E2) (4). E1–E2 heterodimers trimerize into viral spikes that mediate receptor binding and membrane fusion, while the nucleocapsid (NC) encloses the viral RNA genome prior to the glycoprotein envelopment.

Alphavirus particle assembly occurs at the plasma membrane (PM), where spikes progressively assemble while enwrapping underlying NCs. E2–Cp interaction is required, although preformation of cytosolic NCs is not a strict prerequisite to alphavirus assembly and budding (5, 6). Nascent virions pinch-off from the PM once assembly is complete. At the final stage of budding, the membrane scission event to release complete virions was demonstrated to be endosomal sorting complexes required for transport (ESCRT)-independent (7). Our recent cryogenic electron tomography (cryo-ET) and subtomogram averaging (STA) demonstrated that assembly of CHIKV spikes reorganizes cytosolic NCs from nonicosahedral particles into icosahedral cores of the nascent virions (8). The final pinch-off appears to be the speed-limiting step of the dynamic co-assembly of the two-layer icosahedral virion and can leave empty regions termed budding scars on released virions (8).

Increasing evidence suggests alphavirus particles form with a wide range of assembly outcomes. We recently demonstrated that particle morphologies other than the standard *T* = 4 icosahedron were observed in several purified alphaviruses, including a subpopulation of Eastern equine encephalitis virus (EEEV) particles with alternate *T* = 3 icosahedral symmetry (9). The 2D projections of in vitro assembled capsid-like particles from purified Cp of Ross River virus demonstrated disordered components (10). Molecular simulations suggest particle release from membranes can occur without incorporation of the final 1 to 3 spike trimer(s) (11). Cryo-EM reconstructions of purified alphavirus particles typically utilize icosahedral symmetry in data processing and resolutions have been limited to 4–4.5 Å, well below those of virus structures of similar size (12–14). Recently, a block-based reconstruction protocol was developed to correct for the Ewald sphere effects for large virus particles, including Sindbis virus (SINV), Venezuelan equine encephalitis virus (VEEV), and Getah virus (GETV) (15–17), by partitioning an icosahedrally reconstructed map into small blocks for local orientation, contrast transfer function (CTF) refinement, and reconstruction. Such refinement has produced maps with improved final resolutions at ∼2.8–3.5 Å, while also uncovering heterogeneity among the extracted subparticle blocks. None of these structural studies eluded the biological origin, spatial distribution of structural heterogeneity on the virus particle or insights into its potential significance. Better understanding the origin of lack of perfect icosahedral symmetry in alphavirus virion will put the structural asymmetry in the context of the virus assembly mechanism, and thus the therapeutic intervention strategy.

In this study, we developed an image processing protocol to determine the sites of the icosahedral symmetry breakdown in the structure of CHIKV, revealing imperfectly icosahedral particles and multiple conformations of spike assemblies. We also implemented a protocol to circumvent the virion symmetry imperfection and spike structure heterogeneity to enhance the structure resolution of CHIKV spikes to ∼3 Å. The structure allows visualization of interfacial protein–protein interactions and protein contacts with multiple nonproteinous molecules situated within the E1–E2 hydrophobic pocket. A similar data processing protocol was applied to another arthritogenic alphavirus, Mayaro virus (MAYV), confirming symmetry imperfections as a common feature of virions of the *Alphavirus* genus. Our study demonstrates that local imperfections to global icosahedral symmetry in alphavirus originates directly from virus budding and future studies of virus structures and assembly models should take particle imperfection into account.

## Results

### In situ CHIKV virions have a polarized imperfection in icosahedral symmetry

**Fig. 1.**
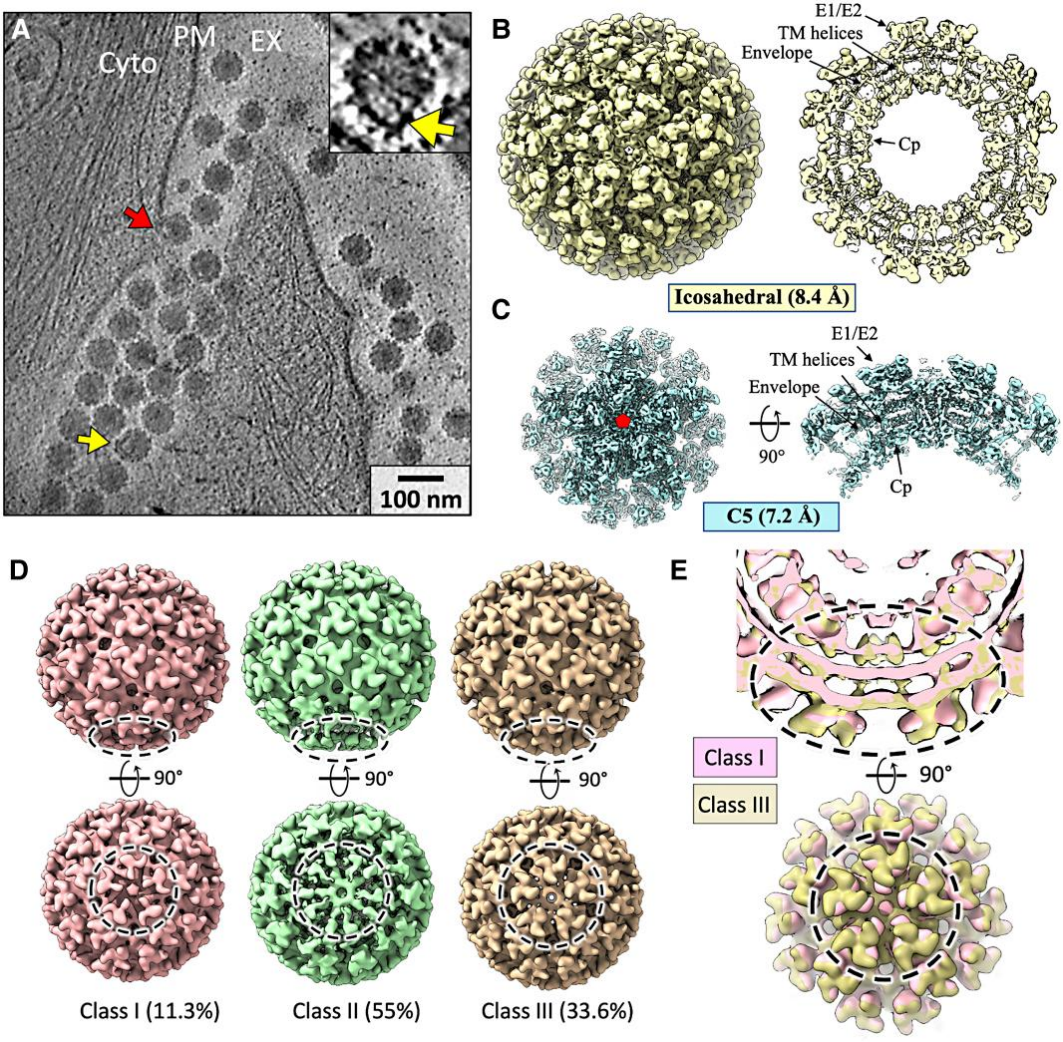
Subtomogram averaged structure of CHIKV virions in situ. A) Tomographic slice view of CHIKV-infected human cells with budding particles along the PM and released particles outside the cell (EX) showing imperfections in icosahedral lattice. EX, extracellular; CYTO, cytoplasm. Inset: zoom-in view of a single released virion with distortions. B) STA of released virion with imposed icosahedral symmetry at 8.4 Å resolution. Central section of density (right) with labeled structural components of virion. C) 7.2 Å STA following focused refinement around penton at a 5-fold axis, with top view (left) and side view (right). D) 3D classes of released virions, with percent of total particles assigned to each class. Poorly resolved pentamer regions displayed with dashed circle. E) Comparison of density at the poorly resolved penton (a dashed circle) between class I and class III. Zoom-in views with half-cut representation (top) and surface view (bottom) shows expansion of class III penton relative to class I.

We recently revealed progressive assembling intermediates of CHIKV in situ using cryo-ET and observed a disordered pole on particles tethered to the PM and released into the extracellular space (8). We hypothesize that the nonicosahedral feature could exist as a feature of released alphavirus virions. In the current study, we first repeated the same cellular cryo-ET analysis of CHIKV-infected human bone epithelial cell (U2OS) and collected tomographic tilt series of the vitrified cells focusing on cell peripheries with nearby released virions. Consistent with our recent report, visual analysis of released virions and nearly fully assembled particles tethered to the cell surface often revealed a region of apparently missing density with a distorted, nonspherical viral envelope that was apparent in 2D slice views in individual particle tomograms (Fig. 1A, Movie S1). Since these individual images have not been subject to STA, such direct visualization directly confirms the presence of nonicosahedral features within some released virions in situ. In addition, these in situ virions were not prepared through in vitro purification, suggesting the nonicosahedral features are not any artifacts associated with sample preparation.

To analyze any consistent nonicosahedral features in these in situ virions, we next analyzed extracellular virions using STA. We extracted 848 CHIKV particles and, initially, applied icosahedral symmetry during averaging to obtain a structure at 8.4 Å resolution that matched reported alphaviruses structure (Figs. 1B and S1A) (13, 18). Focused refinement of subparticle regions, each containing a penton (5 trimers surrounding the icosahedral 5-fold axis) and extra padding density, improved the reconstruction to 7.2 Å resolution (Figs. 1C and S1A, B), suggesting intraparticle heterogeneity exists in the virions. To assess nonicosahedral features, symmetry was relaxed from icosahedral to *C*_5_, revealing a single penton with weaker density. Focused classification around the pentons with less-resolved density resulted in three different classes (Figs. 1D and S1A), each displaying distorted and weaker density of spikes compared with the other 75 spikes of the average. Among the 3D class averages, two different conformations are observed in the penton (class I and class III): class III's spikes are inclined away from the 5-fold vertex, while class I's spikes are closer to the vertex (Fig. 1E, Movies S2 and S3). In class II, the spikes in the disordered penton were not well resolved (Fig. 1D). The expansion/compression of spikes at the penton in each class is matched by Cps below the membrane that forms the NC lattice, supporting our proposed mechanism of spike/Cp lattice co-assembly (8).

The expanded spike conformation (class III) was reminiscent of our recently reported structure of the late stage budding intermediate that has completed assembly and is still connected to the PM (8). To compare the two structures, we superimposed them using the UCSF ChimeraX fit-in-map tool (19) (Fig. S2). The weak density, expanded penton of the released virion map aligns well with the penton connected to PM in the late-stage budding intermediate map and appears expanded and distorted relative to the opposite (leading end) penton (Fig. S2). Taken together, these results suggest that a polarized imperfection within CHIKV particles possibly originates from the assembly/budding process.

**Fig. 2.**
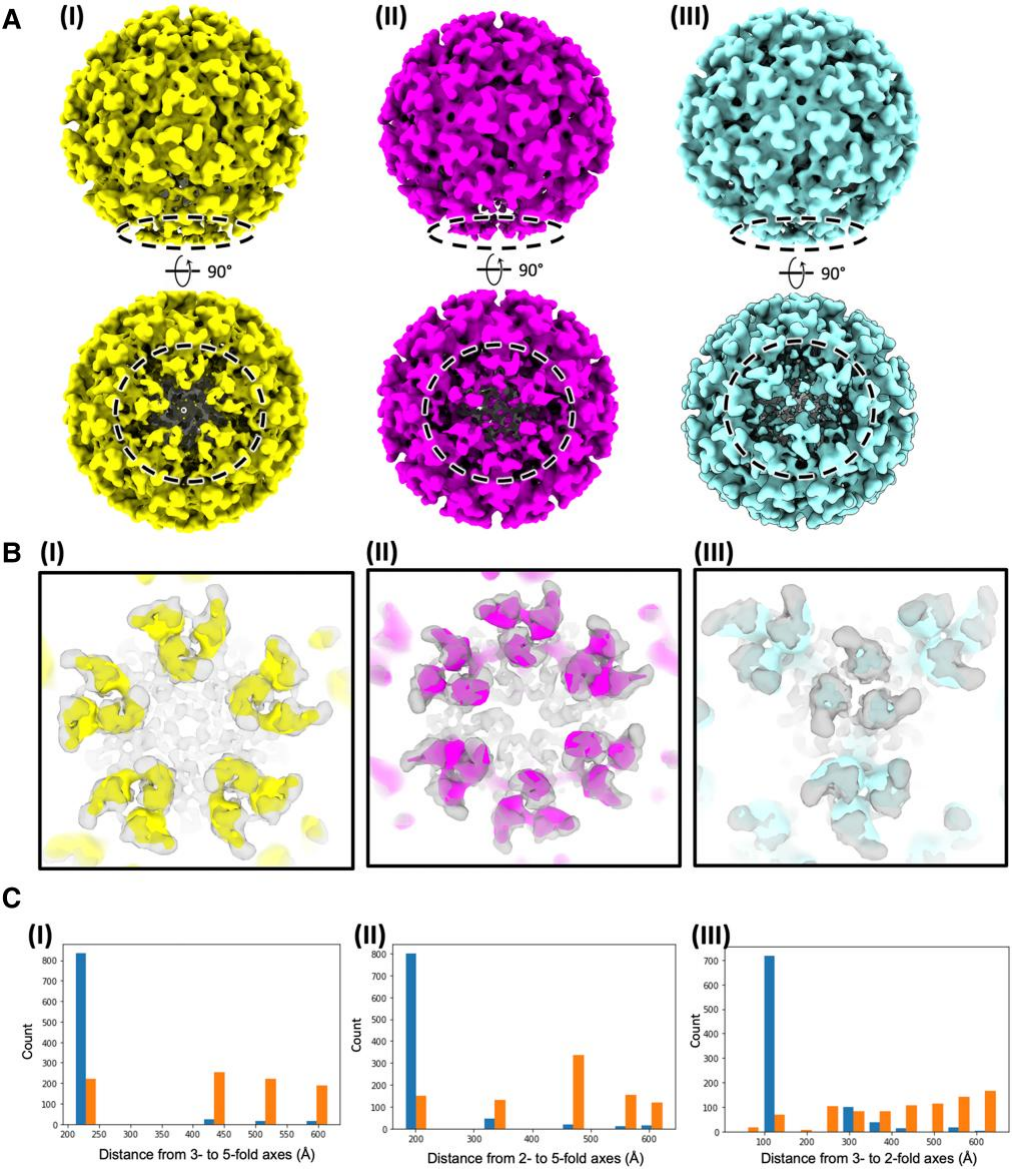
In situ CHIKV virions with imperfect icosahedral symmetry at one pole. A) STAs of icosahedrally averaged virions released the symmetry to (I) *C*_5_ symmetry, (II) *C*_2_ symmetry, or (III) *C*_3_ symmetry. Poorly resolved regions displayed with dashed circle. B) Comparison of the density maps released to (I) *C*_5_ symmetry, (II) *C*_2_ symmetry, or (III) *C*_3_ symmetry with the map with icosahedral symmetry in semi-transparency. C) Histograms of distances from (I) a bad 3-fold axis to a bad 5-fold axis (left) vs. to a random 5-fold axis (right); (II) a bad 2-fold axis to a bad 5-fold axis (left) vs. to a random 5-fold axis (right); and (III) a bad 3-fold axis to a bad 2-fold axis (left) vs. to a random 2-fold axis (right).

To test whether the asymmetric pole is a consistent region that originates from the final pinch-off step of the budding process instead of randomly present imperfections throughout the released virions, we further analyzed the same dataset of in situ CHIKV virions with icosahedral symmetry (Fig. 1B) by releasing either to *C*_2_ or to *C*_3_ symmetry applied (Figs. 2A and S1A). Similar to the results of STA with *C*_5_ symmetry releasing applied (Fig. 2B(I)), distorted spike trimers were observed around a “bad” 2- or 3-fold axis compared with the icosahedral feature in gray (Fig. 2B(II) and (III)). Interestingly, the distances among the bad 2-, 3-, and 5-fold axes to each other predominantly fall in the distances between neighboring axes (Fig. 2C). In marked contrast, the distances from the bad 2- or 3-fold axis to a random 5-fold as well as from the bad 3-fold axis to a random 2-fold axis on the icosahedral virion surface are widely distributed (Fig. 2C). Together, these analyses demonstrated that the disordered 2-, 3-, and 5-fold axes coordinately fall in a same asymmetric pole (Fig. 2C), suggesting the symmetry imperfection is polarized on the virus surface, likely a “scar” left by membrane scission upon assembled particles release from the cell surface.

### In vitro purified infectious alphavirus particles have imperfect icosahedral symmetry

**Fig. 3.**
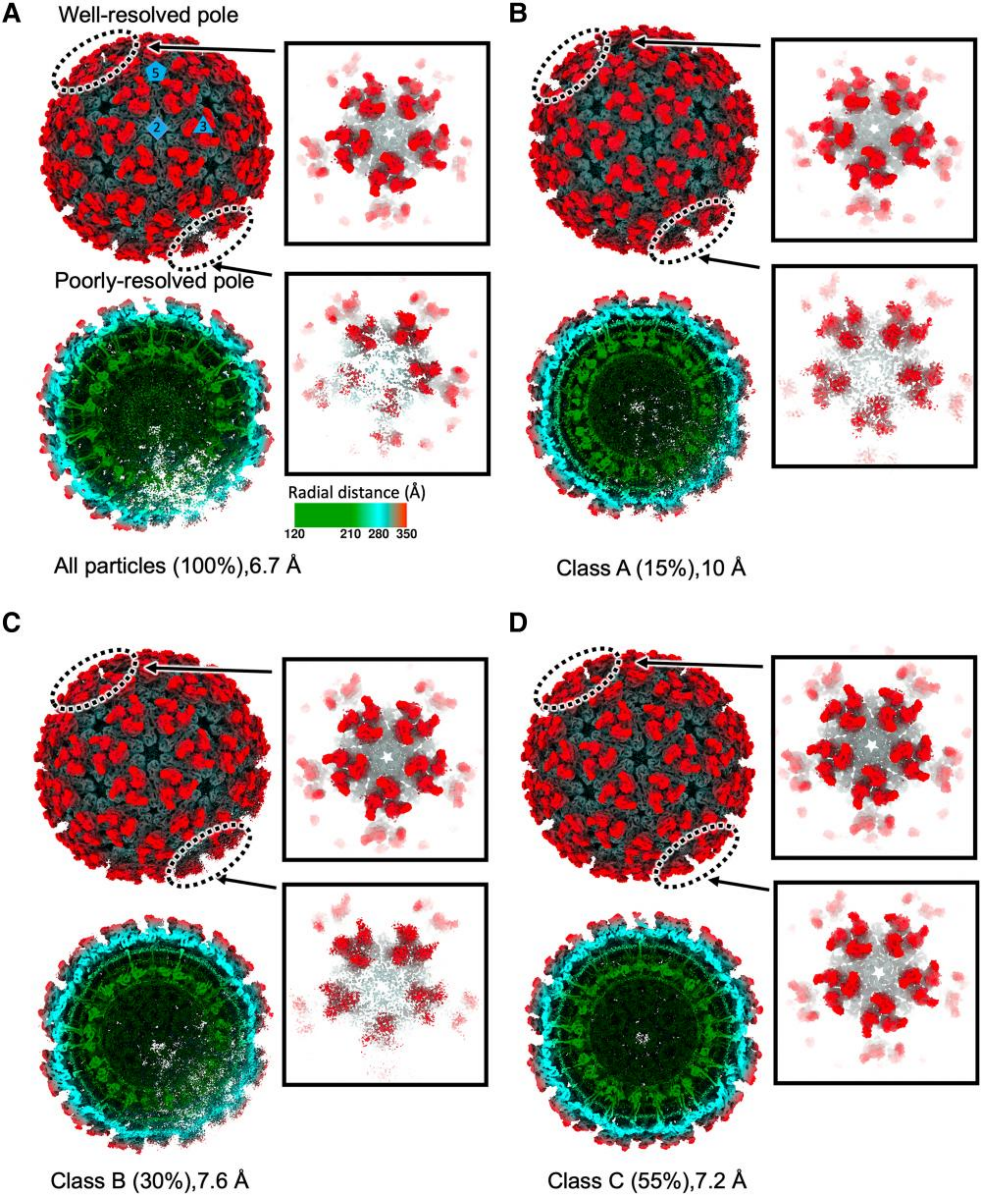
Asymmetric reconstructions of biochemically purified CHIKV particles. A) Radial-colored density maps (above) with half-cut representation (below) following asymmetric (*C*_1_) reconstruction of CHIKV from all particles. Zoom-in views of the well-resolved pole and poorly resolved pole show the two respective pentamers of spikes. Following 3D classification, B) class A, C) class B, and D) class C maps are displayed in the same way as (A), including the entire particle, cross-sectional view, and two penton views with different degrees of 5-fold symmetry appearance. The percentage of particles assigned to each class after 3D classification displayed in parenthesis. All maps are displayed at the same density threshold (3*σ*). RNA density was masked out for visualization.

By analyzing in situ CHIKV virions, we propose the final virus pinch-off from virus-producing cell surface leaves an asymmetric pole on released virions and this inherent structural feature is obscured in classic single-particle analysis of biochemically purified alphavirus particles with icosahedral symmetry applied. To test this hypothesis, we next determined reconstructions of biochemically purified CHIKV particles without imposed symmetry (Figs. 3A and S3A). Infectious CHIKV-181/25 was purified from the supernatant of infected Vero cells, vitrified on a cryo-EM grid, and micrographs were collected using an electron microscope operated at 300 kV (Fig. S3B). From 97,861 total particles identified in the micrographs, an asymmetric *C*_1_ reconstruction of CHIKV was determined at 6.7 Å resolution (Figs. 3A and S3C). The asymmetric map matched the overall architecture of the STA (Fig. 1B) and previously reported icosahedral reconstructions, with equivalent positioning of the spikes and NC core (13, 18). Within the asymmetric reconstruction, one region of the particle most closely matched the icosahedral structure (termed the “well-resolved pole”) in terms of both spike positioning and appearance (Fig. 3A). Directly opposite the well-ordered pole, we observed a region with significant distortions of spike organization, the lipid envelope and Cp organization in the NC core (termed the “poorly resolved pole”; Fig. 3A). Spikes at the poorly resolved pole appear extremely distorted when viewed at a sufficiently high density threshold (Fig. 3A). Therefore, biochemically purified virions possess similar imperfect icosahedral symmetry to in situ virions released from cells grown on the cryo-EM grid.

To further evaluate the asymmetry, all particles were classified without imposed symmetry using RELION 3.1 3D classification (20), resulting in three classes with different degrees of distortion at one pole (Figs. 3B–D and S3A, D, E). Class A (Fig. 3B) and class B (Fig. 3C) maps exhibited the well-resolved pole opposite to the poorly resolved pole found in the reconstruction from all particles (Fig. 3A). In comparison, the entire particle with well-resolved spike structures can be seen in class C, although a higher density threshold is still required to view the slightly less-resolved density pole (Fig. 3D).

To address whether deviations from icosahedral symmetry are found in other alphaviruses, we also collected and processed micrographs of biochemically purified MAYV (strain 12A; Fig. S4A and B), an arthritogenic alphavirus circulating in Central and South America (21, 22). An asymmetric cryo-EM reconstruction of MAYV resolved at 9.5 Å resolution matched the overall organization of CHIKV, with a highly ordered pole showing better-resolved density in both spike and NC layers, and a largely disordered pole directly opposite it (Fig. S4C–G). The similar asymmetry in a separate alphavirus preparation suggests alphavirus virions likely possess a shared imperfect icosahedral particle morphology.

### Asymmetric unit–based analysis to offset imperfection in icosahedral symmetry

**Fig. 4.**
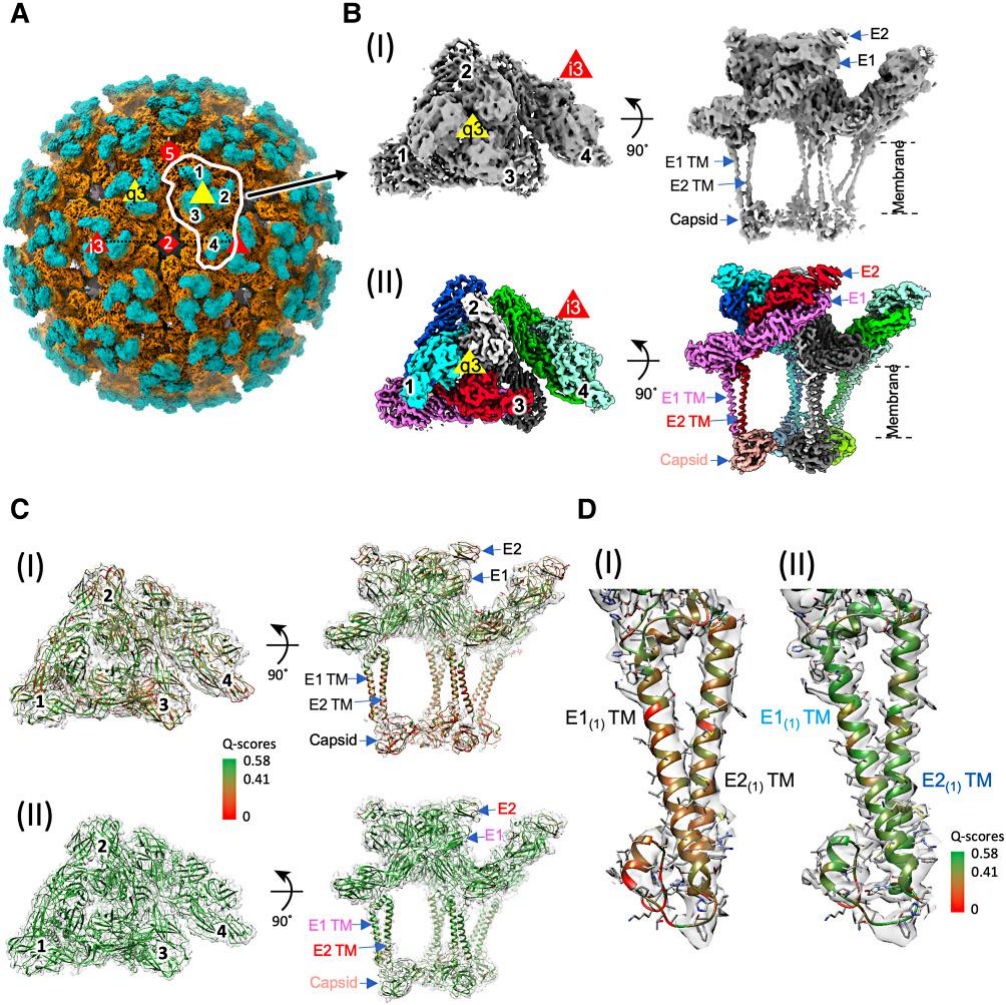
Cryo-EM structure of biochemically purified CHIKV. A) The 4.18 Å resolution radial-colored density map of CHIKV following refinement with icosahedral symmetry imposed. Penton, triangle, and diamond represent 5-, 3-, and 2-fold symmetry axes. An ASU is marked with white outline. The four E1–E2 heterodimers in the CHIKV ASU are labeled with 1–4. B) View of CHIKV ASU after reconstruction (I) icosahedral refinement at ∼4.2 Å and (II) after focused classification and refinement at ∼3.1 Å resolution. C) The ASU model fits in the map (I) before or (II) after additional processing with residues colored by *Q*-scores to show the improvement of map density resolvability. D) Zoom-in view of a single E1_(1)_ and E2_(1)_ with resolved transmembrane helices (TM) displayed with *Q*-scores distribution. Expected *Q*-score values 0.58 and 0.41 correspond to 3 and 4 Å resolution. Subscripts of E1 or E2 represent the numbering of E1–E2 dimer in an ASU in (B).

After confirming the imperfect icosahedral symmetry in biochemically purified CHIKV virions (Fig. 3), we propose that deviations from global symmetry will limit resolution of icosahedral single-particle cryo-EM reconstructions because some asymmetric units (ASUs) are not positioned in the expected icosahedral position or not assembled uniformly and will destructively interfere with the averaging. To test the hypothesis, we carried out additional refinement steps after an initial refinement using RELION 3.1 with imposed icosahedral symmetry that generated a 4.18-Å resolution map using the dataset of 97,861 single-particle images (Figs. 4A and S3A, C, F). Particle orientations were used to expand symmetry and generate the 60 unique ASUs (four E1–E2–Cp subunits) images per particle for further classification analysis (Figs. 4A and S3A). The resulting 5,871,660 ASU subparticles were classified into eight 3D classes of the CHIKV ASU: seven (class 2–8) that range in resolution from 3.4 to 3.9 Å, and an additional low-resolution class (class 1) resolved to 9.2 Å (Fig. S3A).

Atomic models (models 2–8) derived from their corresponding maps better than 4 Å resolution show high overall agreement. The largest differences are observed within the quasi-3-fold (q3) trimer near the 2-fold axis (Fig. S5), specifically in the linker region between E1 domains I and III within q3 E1–E2 heterodimer 2 (Fig. S5). The rmsd of this region between model 2 (reference model) and other models varies from 0.59 Å (model 6) to 4.27 Å (model 3). Due to the close agreement between the models of class average maps 2 and 6 (rmsd 0.59 Å), particles assigned to those classes were pooled together and further refined to produce a map at 3.09 Å (Fig. S3A, C, and F). β-Strands in the E1–E2 ectodomain and helical pitch in transmembrane (TM) helices that were lacking in the initial map became evident (Fig. 4B). The map improvement reveals that focusing data processing on subsets of ASUs in virions with imperfect icosahedral symmetry is necessary to determine the high-resolution map of the structural proteins. This result agrees qualitatively with a separately developed “block-based” reconstruction protocol focusing on larger subparticle regions that was applied to the alphaviruses, such as SINV and GETV (15, 17).

We used the *Q*-scores (23) to measure the resolvability of the observed densities with their fitted CHIKV model (Figs. 4C, S3G, and S6). Q-scores of E1, E2, and Cp residues improved after additional ASU-based processing, particularly for the E1 and E2 TM helices (Figs. 4C, D and S6). The improvement reveals lack of stable spike–Cp interactions in discarded heterogeneous particle regions. Such protein interactions in E1 and E2 TM helices can be described with high confidence. Average *Q*-scores of the spike ectodomains, excluding flexible E2 domain B (E1:0.62; E2:0.64), were significantly higher than the TM helices regions (E1:0.53; E2:0.52) and E2 C-terminus region (E2:0.46). Ectodomain *Q*-score values (excluding flexible domain B) were consistently at values expected in maps at or better than 3 Å, while *Q*-scores of residues of the E1–E2 TM helices and Cp correspond to maps expected between 3 and 4 Å resolution (Figs. 4C, D and S6A). The uneven resolvability of the map is the opposite of typical radial blurring (a geometric artifact caused by data processing and/or angular uncertainty): with radial blurring, ectodomain *Q*-scores would be lower than TM or CP *Q*-scores, but the opposite is seen in this map. Therefore, the CHIKV spike TM helices and Cp are less rigidly organized in the virion relative to the outer spike shell, even in the smallest building block (i.e. ASUs) of the virus particle.

### Intra and inter E1–E2 subunit contacts implicated in alphavirus assembly

**Fig. 5.**
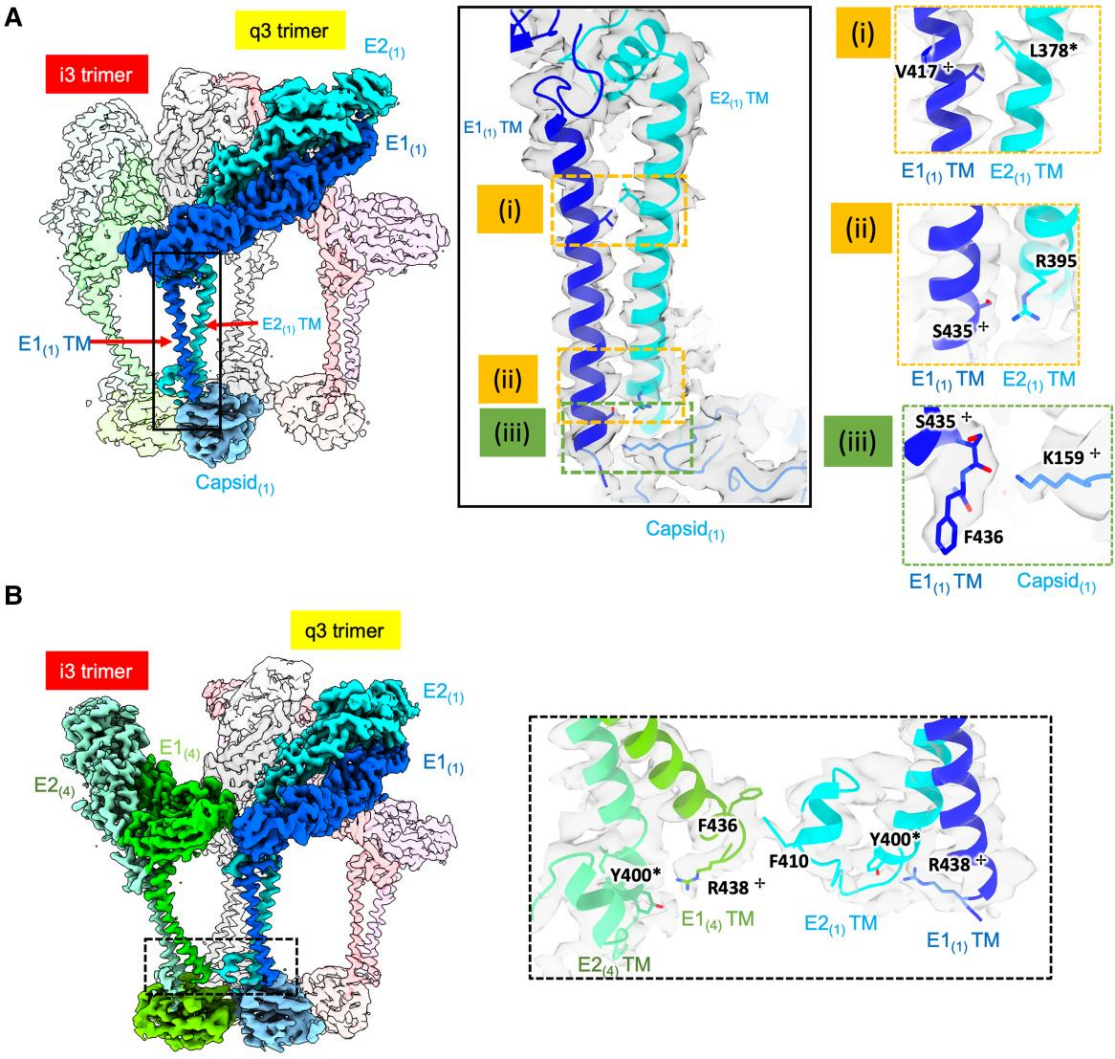
Transmembrane helices and endodomain interactions in biochemically purified CHIKV ASU. A) Multiple interactions of E1 TM region with E2 TM and Cp. Zoom-in views of residues involved in E1_(1)_–E2_(1)_ TM interactions (i, ii), and E1_(1)_–Capsid_(1)_ interaction (iii). B) Interaction across subunits in the q3 spike and neighboring i3 spike. E1_(4)_–E2_(1)_ interaction within q3-i3 spike displayed as zoom-in views. Residues labeled with a cross or star indicate highly^✢^/completely* conserved residues across different alphaviruses in Fig. S7. Each protein molecule is colored uniquely and matches colors in Fig. 4B(II).

In a previously reported CHIKV p62-E1 ectodomain crystal structure, extensive E1–E2 ectodomain contacts were identified (24), but details of the CHIKV TM domain and endodomain were missing. Our ASU model reveals additional putative residue contacts within the lipid bilayer region between E1 and E2 TM helices and Cp. Many of the identified residues show high sequence conservation with other alphaviruses (Figs. 5 and S7). Below the characteristic E1 TM kink region, contacts begin at E1 Val417 and E2 Leu378 (Fig. 5A(i)) and continue to the base of the TM helices. Near E1 C-terminus, putative hydrogen bonds exist between E1 Ser435 and E2 Arg395 (Fig. 5A(ii)), and E1 Arg438 and E2 Tyr400 (Fig. 5B). The proximity of these interactions to the E2 C-terminus suggests that they may contribute to the stabilization of the E2 C-terminal tail conformation that makes extensive contacts with Cp. While E2 C-terminal tail and Cp interaction was reported as critical to virus assembly/budding based on mutation experiment (25), our structure shows that E1 C-terminal residues also contact Cp. This intermolecular interface also includes a putative hydrogen bond between Cp Lys159 and E1 main chain Ser435 carboxyl group (Fig. 5A(iii)). Anchoring of E1 C-terminus by Cp contacts could also stabilize both E1 and E2 TM conformations that promote spike-capsid co-assembly.

Lateral interactions between q3 and i3 trimers are well known to form the *T* = 4 icosahedral surface lattice through E1–E1 ectodomain interactions (5). Hereby, we annotate the E1–E2 dimers around the q3 in subscripts 1, 2, and 3, and the dimer around i3 in subscript 4 (Figs. 4A and 5A). Our atomic model reveals extra contacts between the E1_(1)_–E2_(1)_ TM region and the neighboring E1_(4)_–E2_(4)_ TM region at the q3-i3 trimer interface. Phe436 of E1_(4)_ at the base of E1 TM helix contacts Phe410 of E2_(1)_ in the E2 endodomain tail through a putative π–π interaction (Fig. 5B). Next to E2 Phe410 is the previously reported short helix that loops back to the inner membrane and anchors the E2 cytoplasmic tail (12). This interaction could assist in properly orienting the C-terminal tails of both E1 and E2 for spike–Cp interactions.

In addition to the interactions between E1 and E2 TM helices of neighboring E1–E2 heterodimers in CHIKV ASU (Fig. S8A), a considerable conformational difference exists between the TM region of one E1–E2 dimer 3 and the other three E1–E2 dimers 1, 2, and 4 in the ASU (Fig. S8B). The rmsd of this TM region between E1_(1)_–E2_(1)_ and E1_(3)_–E2_(3)_ is 4.1 Å for E1 TM and 5.6 Å for E2 TM, respectively. From the contacts of envelope protein and NC in virion structure, E1–E2 dimer 3 in the ASU contacts a Cp molecule that is part of a NC pentamer, while E1–E2 dimers 1, 2, and 4 contact Cps of an NC hexamer. In addition to the TM region being the most flexible part of the glycoprotein structure (Figs. S6 and S8B), the multiple conformations suggest it can provide plasticity that facilitates co-assembly of spike and capsid lattices.

### Posttranslational modification and lipid-like molecules identified in the CHIKV spikes

Glycans posttranslationally attached on alphavirus envelope proteins E1–E2 are reported to bind host cell receptors and determine viral pathogenesis in vivo (26, 27). CHIKV E1–E2 has three predicted N-linked glycosylation sites, at E1 Asn141, E2 Asn263, and Asn345. We confirmed the presence of extra density corresponding to N-linked glycan modifications at each predicted site in CHIKV map (Fig. S9).

**Fig. 6.**
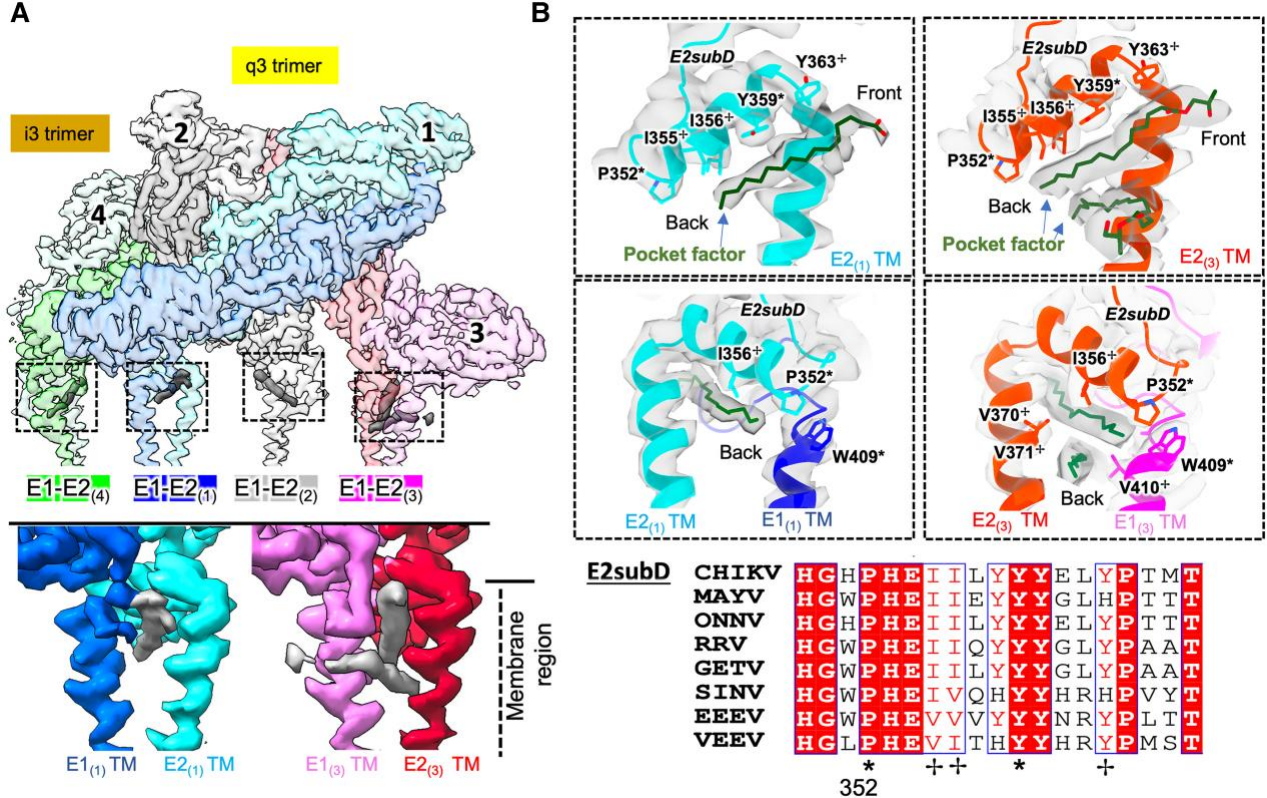
Lipid-like densities within the E1–E2 dimer of CHIKV. A) CHIKV ASU map highlights the positions of pocket factor densities (dashed squares). Two densities were found in the E1_(3)_–E2_(3)_ dimer (bottom right) and one density in other dimers (bottom left). B) Zoom-in model views of E1–E2 hydrophobic pockets, with key residues forming interactions with the pocket factor labeled. The alkyl chain models of phospholipid fit into the extra densities in the CHIKV E1–E2 hydrophobic pocket. Residues labeled with a cross, or star are highly^✢^/completely* conserved residues across different alphaviruses. E2subD:E2 subdomain D.

A potential lipid “pocket factor” was reported for different alphaviruses after the observation of unidentified, single linear densities in an E1–E2 pocket near the viral membrane (12, 14–17). In our CHIKV ASU map, E1–E2 dimers 1, 2, and 4 have a single linear density in the hydrophobic pocket ∼17 Å long that sits below E2 subdomain D and runs roughly parallel to its length, and E1–E2 dimer 3 in the q3 trimer has two distinct pocket factor densities with the top one equivalent to the ones in other three E1–E2 dimers (Figs. 6 and S10A). The hydrophobic phospholipid tail of phosphatidylcholine was built into the pocket factor density of each subunit (Figs. 6B and S10B). *Q*-scores of the phospholipid tail model in the pocket factor density of E1–E2 dimers 1, 2, and 4 were 0.55, 0.67, and 0.62, which is close to expected *Q*-scores at 3 Å. The highest *Q*-scores of the phospholipid tail models found in E1–E2 dimer 3 were 0.72 (top) and 0.71 (bottom). The CHIKV cavity with a volume around ∼1,300 Å^3^ is larger than other alphaviruses (MAYV, SINV, EEEV, and VEEV: an average volume ∼800 Å^3^) because E1 and E2 TM helices of CHIKV above the kink region are more distant from each other (14). Both pocket factors are apparently stably bound based on a similar viewing density threshold to surrounding protein and high *Q*-scores (Fig. 6). The top pocket factor with linear shape contacts Tyr359 and Tyr363 of E2 subdomain D (Fig. 6B) and is oriented below and roughly parallel to the short alpha helix of E2 subdomain D (Fig. 6B). The posterior of the top pocket factor is surrounded by Ile355, Ile356, Pro352 of subdomain D of E2 and Trp409 of E1 (Fig. 6B). The bottom pocket factor contacts Val370 and Val371 of E2 and Val410 of E1 (Fig. 6B). Multisequence alignment with other alphaviruses revealed these putative pocket factor interacting residues are evolutionarily conserved (Figs. 6B and S7).

## Discussion

Recently, a “block-based” reconstruction method was developed to compensate for the Ewald sphere effect in single-particle analysis of large complexes (28). This method has successfully been applied to extend the resolution of the reconstructions of alphaviruses, including SINV (15), VEEV (16), and GETV (17) to ∼3 Å. While this manuscript was in preparation, a study of CHIKV-like particle (VLP) vaccine was published (29). Using ASU-based reconstruction, the authors reported antibody Fab-bound CHIKV VLP structure at ∼3 Å, although the origin and characteristics of the heterogeneity among ASUs within one VLP particle were not discussed in that study.

In this study, we detail the imperfect icosahedral organization of alphavirus virions at one pole with an integrative cryo-EM approach directly linking structural observations to underlying virus assembly in situ. This observation was made in two different viruses, with different imaging and computational workflows, indicating the structural feature is an inherent characteristic of the alphavirus particle rather than an artifact of specimen preparation or method of image analysis. STA analysis of in situ CHIKV freshly released from virus-infected cells grown on the EM grids demonstrated a disordered end no matter whether *C*_2_, *C*_3_, or *C*_5_ symmetry was imposed (Fig. 2). More interestingly, the disordered or bad 2-, 3-, and 5-fold axis densities are close to each other instead of placed randomly on the virion surface, suggesting a consistent, localized imperfection on in situ CHIKV virions. We previously observed late-stage CHIKV budding particles on the PM with near-complete budding shells and expanded penton at the trailing end (8), which structurally is consistent with one class of in situ released CHIKV. This indicates that the single disordered pole on released virion originates from membrane scission at the last step of virus budding, where a budding scar is left on released particle. We therefore observe the budding scar both directly before (8) and after release. Imperfectly assembled particles at the trailing end of icosahedral virus budding should promote more efficient virion release from cell membranes, in accordance with reported molecular simulations that detail steric clashes between spikes and limited spike diffusion at the latest budding stages with high negative Gaussian curvature of the budding neck (30). The budding scar is consistent with previous reports of imperfect icosahedral symmetry within a purified virus of the Flaviviridae family and can be a common structural feature of spherical enveloped virus assembly that releases from host membrane surface (31).

Alphavirus budding is ESCRT independent (7) and its productive late-stage budding intermediates are strikingly different from ESCRT-dependent HIV budding intermediates. It was reported that the HIV budding is initially promoted by Gag assembly and upon ½–¾ completion ESCRT mediates the membrane scission and virus release (32). This leaves a big gap and multiple defects in assembled hexameric gag lattice in the immature HIV particles (33). During virus maturation, the HIV gag lattice undergoes a large-scale rearrangement. In contrast, alphavirus assembly is driven by the lateral spike–spike and vertical spike–Cp interactions where nearly complete assembly intermediates finally pinch-off the PM in an ESCRT-independent way leaving a small scar on the released virion at the position of the membrane budding neck. This difference between alphavirus and retrovirus budding scar may reflect different assembly mechanisms between double protein shells (alphavirus) and one protein shell (retrovirus) as well as different membrane scission mechanisms.

Multiple conformations and weak density of spike assemblies within the budding scar of CHIKV virions is consistent with hypotheses that spike incorporation into the icosahedral lattice causes a conformational change or rigidifies the spikes which eliminates accessibility of spike epitopes (34, 35). It may also suggest that even with a small budding scar, alphaviruses undergo some rearrangement of the spike and NC shells after virus release. The spikes and Cps in the expanded penton at the disordered pole may undergo some conformational changes to fill in the empty space to better protect the viral genome enclosed inside. This is in marked contrast to the maturation of flaviviruses that have immature and mature glycoproteins incorporated and the glycoprotein reorganization on the chimeric particles is not synchronized (36). Looser packing of spikes at the pole with less interfacial constraints and Cp contacts can also be expected to undergo the pH-mediated membrane fusion cascade more readily during virus entry.

Understanding viral structural heterogeneity and incorporating it into our analyses of virus structures is crucial for gaining detailed structures at a higher resolution. In this study, we have uncovered previously undocumented E1–E2 TM helix interactions. This explains the previous reports of budding defects of viruses carrying mutations at E1 TM helix (37–39), supporting the critical role of E1–E2 interactions around the TM region for alphavirus assembly/budding. Our study demonstrates that E1 contributes to spike–Cp co-assembly not only through direct interactions with Cp but also by assisting in the positioning of the E2 endodomain, which inserts into the hydrophobic pocket on the Cp surface that was believed to be the main vertical spike–Cp interactions (40). Additionally, our CHIKV ASU map has unveiled distinctive double lipid-like pocket factors inserted into the notably large hydrophobic pocket formed by the membrane-proximal regions of CHIKV E1–E2 TM helices. This is in contrast to other alphaviruses, which have a narrower hydrophobic pocket with one lipid-like factor inserted (12, 14–17). The presence of these lipid-like pocket factors suggests a potential role in stabilizing the assembled E1–E2 structure and participating in virus assembly, reminiscent of membrane-proximal lipid-like factors reported for flaviviruses (41–43). While the timing and mechanism of lipid-like factor insertion into the glycoprotein lattice remain unknown for both flaviviruses and alphaviruses, the effective anti-ZIKV function of lipid-targeting agents (44) suggests a similar future drug design can be applicable to alphaviruses. Since the identified E1–E2 residues interacting with the pocket factor(s) are highly conserved across different alphaviruses, the conserved E1–E2 pocket factor interfaces can potentially be targets for broad antivirals. Ultimately, we expect the reported alphavirus structural details will be useful for future design of antiviral therapeutics and provide insights to understand alphavirus biology.

## Materials and methods

### Cryo-ET and subtomogram analysis of CHIKV-infected cells

U2OS human osteosarcoma cells and BHK21 hamster fibroblast cells were purchased from ATCC (cat. no. HTB-96 and CCL-10, respectively). Cells were maintained at 37 °C with 5% humidified CO_2_ in DMEM (Invitrogen) supplemented with penicillin and streptomycin, 10 mM N-2-hydroxyethylpiperazine-N-2-ethane sulfonic acid (HEPES), nonessential amino acids, and 10% fetal bovine serum (FBS) (Hyclone). CHIKV vaccine strain 181/clone 25 (CHIKV 181/25) was amplified in BHK21 cells. U2OS cells grown on fibronectin-coated gold 200 mesh R2/2 grids (Quantifoil), as previously described (8), were infected with CHIKV-181 at a multiplicity of infection of 50 for 8 h. Grids were then washed with phosphate-buffered saline (PBS), and a solution of 10 nm bovine serum albumin (BSA) gold tracer (cat. #25486, EMS) was added directly prior to vitrification. Grids were blotted and plunged into liquid ethane using a Leica EM-GP plunge freezer and were subsequently stored under liquid-nitrogen conditions until data collection.

Grids of vitrified virus-infected cells were imaged on a Titan Krios (Thermo Fisher) electron microscope operated at 300 kV with postcolumn energy filter (20 eV slit width) and K2 Summit detector with calibrated pixel size of 2.72 Å. Images were collected using a Volta phase plate. Single-axis, bidirectional tilt series were collected using SerialEM software with low-dose settings and defocus range of −3 to −5.5 µm. A total average dose of 120 e^−^/A^2^ was applied at the specimen, distributed over 51 tilt images covering an angular range of −50° to +50°, with an angular increment of 2°. The motion between frames of each tilt image in the tilt series was corrected using MotionCor2 software (45). Tilt images were compiled, automatically aligned, and reconstructed into tomograms using EMAN2 software (46).

The 848 released CHIKV particles were selected and extracted into subvolumes. To generate an initial model, 50 high-SNR particles in different orientations were picked from tomograms at 4× binning. These particles were input to the EMAN2 initial model generation program, performed in two steps. First, particles were iteratively aligned with *C*_1_ symmetry for three iterations and aligned to the 5-fold symmetry axis. Then, an additional five iterations were performed, applying *C*_5_ symmetry. This initial model was filtered to 40 Å resolution and used for subsequent 3D subtomogram refinement of the full dataset with icosahedral (icos) symmetry. Subtomogram refinement, followed by subtilt refinement of particle translation and rotation, resulted in a converged 3D reconstruction at 8.4 Å resolution (0.143 Fourier shell correction [FSC] gold-standard criterion). From the icosahedral refinement of the virion, unbinned subparticles were extracted at each penton. Local subtomogram refinement, subtilt translation, and rotation refinement, as well as defocus refinement, were performed on the subparticles, resulting in a penton structure at 7.2 Å resolution. The refinements were performed with *C*_5_ symmetry, and the even/odd split of the virion particles was maintained to avoid overestimation of resolution.

To classify the subtomograms, we first performed iterative symmetry releasing from the icosahedral refinement to *C*_5_ symmetry, using a large, soft mask focusing on one penton. This led to a virion structure with weaker density on one side. Then, iterative 3D classifications were performed from the *C*_5_ refinement, resulting in the three classes shown in Fig. 1.

To investigate the spatial correlation between the 5-, 3-, and 2-fold axes, we started from the icosahedral refinement, and performed iterative symmetry releasing to 3- and 2-fold axes, using the same protocol as the *C*_5_ symmetry releasing described above. Both refinements showed virion structures with weaker density on one of the corresponding symmetry axes. Then, for each virion, we measured the distance from the 5-fold axis of weak density to the 2- and 3-fold axes of weak densities and plotted the histogram of the distances for all virions (Fig. 2). For comparison, we also show the distance from the 2- or 3-fold axes of weak densities to a random 5-fold axis for each particle. This clearly indicates the bad units form clusters, instead of distributing randomly on the virion.

### CHIKV cryo-EM sample preparation and data acquisition for single-particle analysis

CHIKV vaccine strain 181/clone 25 was obtained through BEI Resources, NIAID, NIH (cat. no. NR-13222). CHIKV 181/25 was collected from the supernatant of infected Vero cells as described previously (47). Quantifoil R 2/1 carbon 200 mesh grids with a thin layer of carbon were used for specimen freezing. EM grids were glow discharged for 25 s using a PELCO EasiGlow. Three microliters of sample were applied to each grid and incubated for 30 s. After incubation, the grids were blotted for 2 s at ∼100% humidity and 20 °C and then plunge frozen in liquid ethane with a Leica EM GP.

A total of 6,616 micrographs were then collected on a Titan Krios electron microscope (Thermo Fisher) operated at 300 kV under liquid-nitrogen temperature. Movies were recorded with the software EPU (Thermo Fisher) using a K2 Summit direct-electron detector (Gatan) equipped with GIF quantum energy filter (20 eV; Gatan) in counted mode (pixel size: 1.34 Å/pixel). Each micrograph was exposed for 9 s with 5.25 e^−^/Å^2^/s dose rate (total specimen dose: 47.25 e^−^/Å^2^), and 40 frames were captured per micrograph.

### CHIKV cryo-EM data processing

All micrographs were imported into RELION 3.1 (20) for image processing and 3D reconstruction. The motion correction was performed using MotionCor2 (45) and the CTF was determined using CTFFIND4 (48). By using the Laplacian-of-Gaussian algorithm, 142,609 particles were picked from the micrographs and subjected to 2D classification. The poor 2D class averages not showing good protein features were then removed following five rounds of 2D classification.

For the asymmetric reconstruction of the whole virus map, 97,861 particles selected after 2D classification were subjected to ab initio model generation with *C*_1_ symmetry and submitted to 3D autorefinement with *C*_1_ symmetry imposed. To classify the virus particles with *C*_1_ symmetry, the particles selected after 2D classification were subjected to 3D classification with *C*_1_ symmetry with three output classes. The three resulting classes were then subjected to 3D autorefinement with imposed *C*_1_ symmetry. For the icosahedral map, the same particles selected after 2D classification were subjected to ab initio model generation with icos symmetry (RELION I4 setting) imposed and then submitted to 3D autorefinement with imposed icos symmetry. Then CTF refinement was performed, and the particles after CTF refinement were subjected to 3D autorefinement with icos symmetry, resulting in a map at 4.18 Å resolution estimated using the gold-standard FSC = 0.143 criterion (49). For focused classification of ASUs, the orientation of each ASU was first determined using the “relion_particle_symmetry_expand” command (50) following the 3D autorefinement of virus particles with icos symmetry. This computed the rotational matrix to determine the location and orientation for each of the 60 ASUs in each original virus particle image, based on icosahedral symmetry. Sixty subparticle images were output for each virus particle image with each of the 60 ASU subparticles aligned in the same orientation. We created a mask around the ASU using the Segger tool in UCSF Chimera (51). Masked subparticles (5,871,660 particles) following symmetry expansion were subjected to focused 3D classification without image alignment and *C*_1_ symmetry with 8 output classes. The subparticles within each of the eight 3D classes were subjected to 3D autorefinement without imposed symmetry. The same 3D mask was used in each case and the respective class average served as the initial model. The maps after 3D autorefinement were used to build atomic models for the comparison described in the “CHIKV model building, refinement, and analysis” section. Particles of class 2 and class 6 were combined into a group with 2,252,668 particles for a further 3D autorefinement with *C*_1_ symmetry. CTF refinement was then performed, refining magnification anisotropy, optical aberrations (up to the 4th order), and finally refining per-particle defocus and per-micrograph astigmatism. The particles after CTF refinement were subjected to a final focused 3D autorefinement with *C*_1_ symmetry. This yielded a final map resolved at 3.09 Å resolution. Global resolutions were estimated using the gold-standard FSC = 0.143 criterion (49) after applying a soft mask around the protein density created by RELION 3.1.

### CHIKV model building, refinement, and analysis

The structure of the ASU containing E1, E2, and Cps from the CHIKV model (PDB:6nk5) was rigidly fitted to the class 2 ASU map. Individual chains were then interactively refined using ISOLDE (52) to match the density. After fitting with ISOLDE, the ASU model was refined using MDFF (53) and then phenix.real_space_refine (54) iteratively. After comparing models built in a similar way in maps for classes 2 to 8, it was determined that models for classes 2 and 6 were very similar, and, hence, the particles of classes 2 and 6 were combined to generate the map with improved resolution of 3.09 Å. Then, the final model was refined into the resulting 3.09 Å map. Segments for pocket factors were identified using Segger. For docking to these segments, lipids were modeled using phenix.elbow (using chemical_component PCW parameter) and fit to the segments using SegFit. The structure of each lipid was interactively refined using Chimera and SegMod to better fit the observed densities. The final model was again refined using phenix.real_space_refine. Refinement statistics of the structural model are listed in Table S1. The local resolution was estimated by ResMap (55). *Q*-scores (23) were used to evaluate map-model agreement. Isosurfaces and models were visualized with UCSF Chimera (56) or ChimeraX (19). The figures for electrostatic view were made by APBS Electrostatics in PyMOL (v.2.2 Schrodinger, LLC).

### Mayaro virus purification

Vero cells were prepared to 80–90% confluence and inoculated with MAYV strain 12A at a multiplicity of 0.1 plaque-forming units per cell. Infected cells were incubated at 37 °C for 2 days or until cytopathic effects were observed (57). Cellular debris was removed from the culture supernatant by centrifugation for 5–10 min at 1,000–2,000 × *g*. Virus was concentrated by precipitation with 7% polyethylene glycol 6,000 and 2.3% NaCl at 4 °C for 12 h. Virus was then pelleted by centrifugation at ⩾3,100 × *g* for 30 min and gently resuspended in 2 mL tris-EDTA-Nacl (TEN) buffer (0.05 M Tris–HCl, pH 7.4, 0.1 M NaCl, and 0.001 M ethylenediaminetetraacetic acid (EDTA)). The virus suspension was purified by centrifugation through a 20–70% continuous sucrose/TEN gradients for 60 min at 210,000 × *g*. The virus band was harvested and centrifuged 5× through an Amicon 100 kDa filter (Ultra-4, cat. no. UFC810024), resuspending each time to maximum load volume with TEN. The purified virus was harvested in the minimal remaining volume after final centrifugation.

### Mayaro virus cryo-EM sample preparation, data acquisition, and data processing for single-particle analysis

The preparation of MAYV particles was applied to Quantifoil copper EM grids with a holey-carbon film and plunge frozen in liquid ethane using a FEI Vitrobot Mark IV freezing apparatus. The frozen-hydrated MAYV grids were examined with a JEM-3200FSC microscope, operated at liquid-nitrogen temperature, and equipped with a Gatan K2 Summit direct-electron detector. Images were collected in super-resolution mode at 30,000× magnification and binned by 2 for processing of images at a pixel size of 1.28 Å. Each image exposure was for 5 s, with an electron dose rate of 7 electrons/pixel/second, resulting in a dose of 35 electrons on the specimen over a total of 25 frames. In total, ∼1,100 images were collected, and 22,314 particle images were selected for further processing.

An asymmetric reconstruction of MAYV using the 22,314 selected particles was performed using RELION 3.0 (20) using *C*_1_ symmetry. Prior to refinement, micrographs were further binned to a pixel size of 2 Å, motion-corrected using MotionCor2, and CTF parameters were estimated using CTFFIND4 (45, 48). An icosahedrally symmetric starting model of MAYV was low-pass filtered to 60 Å to blur the high-resolution features. Orientations of each particle were determined without imposing symmetry, with global angular search value of 3.7° and local search angular increment of 0.9°. The orientation search converged after 26 iterations, resulting in an asymmetric reconstruction at ∼9 Å resolution using gold-standard FSC criteria. The figures were prepared using UCSF Chimera (56) or UCSF ChimeraX (19).

## Supplementary Material

pgae102_Supplementary_Data

## Data Availability

The STA cryo-EM maps of CHIKV virion are deposited in the wwPDB OneDep System under EMD accession codes EMD-41096 (focused refinement). The cryo-EM map of the CHIKV ASU, with its associated atomic model, has been deposited under EMD accession code EMD-28979 and PDB ID code 8fcg. The cryo-EM maps of CHIKV and MAYV with asymmetric reconstruction have been deposited under EMD accession code EMD-41631 and EMD-41637.

## References

[1] Strauss JH, Strauss EG. 1994. The alphaviruses: gene expression, replication, and evolution. Microbiol Rev. 58:491–562. 7968923 10.1128/mr.58.3.491-562.1994 PMC372977

[2] de Lima Cavalcanti TYV, Pereira MR, de Paula SO, Franca RFdO. 2022. A review on chikungunya virus epidemiology, pathogenesis and current vaccine development. Viruses. 14(5):969. 35632709 10.3390/v14050969 PMC9147731

[3] Bartholomeeusen K, et al 2023. Chikungunya fever. Nat Rev Dis Primers. 9:17. 37024497 10.1038/s41572-023-00429-2 PMC11126297

[4] Button JM, Qazi SA, Wang JC-Y, Mukhopadhyay S. 2020. Revisiting an old friend: new findings in alphavirus structure and assembly. Curr Opin Virol. 45:25–33. 32683295 10.1016/j.coviro.2020.06.005 PMC7746636

[5] Forsell K, Xing L, Kozlovska T, Cheng RH, Garoff H. 2000. Membrane proteins organize a symmetrical virus. EMBO J. 19:5081–5091. 11013211 10.1093/emboj/19.19.5081 PMC302099

[6] Zheng Y, Kielian M. 2015. An alphavirus temperature-sensitive capsid mutant reveals stages of nucleocapsid assembly. Virology. 484:412–420. 26051211 10.1016/j.virol.2015.05.011 PMC4567448

[7] Taylor GM, Hanson PI, Kielian M. 2007. Ubiquitin depletion and dominant-negative VPS4 inhibit Rhabdovirus budding without affecting alphavirus budding. J Virol. 81:13631–13639. 17913808 10.1128/JVI.01688-07 PMC2168838

[8] Chmielewski D, Schmid MF, Simmons G, Jin J, Chiu W. 2022. Chikungunya virus assembly and budding visualized in situ using cryogenic electron tomography. Nat Microbiol. 7:1270–1279. 35773421 10.1038/s41564-022-01164-2 PMC9930444

[9] Kaelber JT, Chmielewski D, Chiu W, Auguste AJ. 2022. Alphavirus particles can assemble with an alternate triangulation number. Viruses. 14:2650. 36560655 10.3390/v14122650 PMC9780915

[10] Wang JC-Y, Chen C, Rayaprolu V, Mukhopadhyay S, Zlotnick A. 2015. Self-assembly of an alphavirus core-like particle is distinguished by strong intersubunit association energy and structural defects. ACS Nano. 9:8898–8906. 26275088 10.1021/acsnano.5b02632 PMC5683390

[11] Lázaro GR, Mukhopadhyay S, Hagan MF. 2018. Why enveloped viruses need cores—the contribution of a nucleocapsid core to viral budding. Biophys J. 114:619–630. 29414708 10.1016/j.bpj.2017.11.3782 PMC5985022

[12] Zhang R, et al 2011. 4.4 Å cryo-EM structure of an enveloped alphavirus Venezuelan equine encephalitis virus. EMBO J. 30:3854–3863. 21829169 10.1038/emboj.2011.261 PMC3173789

[13] Basore K, et al 2019. Cryo-EM structure of chikungunya virus in complex with the Mxra8 receptor. Cell. 177:1725–1737.e16. 31080061 10.1016/j.cell.2019.04.006 PMC7227486

[14] Ribeiro-Filho HV, et al 2021. Cryo-EM structure of the mature and infective Mayaro virus at 4.4 Å resolution reveals features of arthritogenic alphaviruses. Nat Commun. 12:3038. 34031424 10.1038/s41467-021-23400-9 PMC8144435

[15] Chen L, et al 2018. Implication for alphavirus host-cell entry and assembly indicated by a 3.5Å resolution cryo-EM structure. Nat Commun. 9:5326. 30552337 10.1038/s41467-018-07704-x PMC6294011

[16] Ma B, Huang C, Ma J, Xiang Y, Zhang X. 2021. Structure of Venezuelan equine encephalitis virus with its receptor LDLRAD3. Nature. 598:677–681. 34646021 10.1038/s41586-021-03909-1

[17] Wang A, et al 2022. Structure of infective Getah virus at 2.8 Å resolution determined by cryo-electron microscopy. Cell Discov. 8:12. 35149682 10.1038/s41421-022-00374-6 PMC8832435

[18] Sun S, et al 2013. Structural analyses at pseudo atomic resolution of Chikungunya virus and antibodies show mechanisms of neutralization. Elife. 2:e00435. 23577234 10.7554/eLife.00435 PMC3614025

[19] Goddard TD, et al 2018. UCSF chimerax: meeting modern challenges in visualization and analysis. Protein Sci. 27:14–25. 28710774 10.1002/pro.3235 PMC5734306

[20] Zivanov J, et al 2018. New tools for automated high-resolution cryo-EM structure determination in RELION-3. Elife. 7:e42166. 30412051 10.7554/eLife.42166 PMC6250425

[21] Anderson CR, et al 1957. Mayaro virus: a new human disease agent. II. Isolation from blood of patients in Trinidad, B.W.I. Am J Trop Med Hyg. 6:1012–1016. 13487973 10.4269/ajtmh.1957.6.1012

[22] Lorenz C, Freitas Ribeiro A, Chiaravalloti-Neto F. 2019. Mayaro virus distribution in South America. Acta Trop. 198:105093. 31325416 10.1016/j.actatropica.2019.105093

[23] Pintilie G, et al 2020. Measurement of atom resolvability in cryo-EM maps with Q-scores. Nat Methods. 17:328–334. 32042190 10.1038/s41592-020-0731-1 PMC7446556

[24] Voss JE, et al 2010. Glycoprotein organization of chikungunya virus particles revealed by X-ray crystallography. Nature. 468:709–712. 21124458 10.1038/nature09555

[25] Skoging U, Vihinen M, Nilsson L, Liljeström P. 1996. Aromatic interactions define the binding of the alphavirus spike to its nucleocapsid. Structure. 4:519–529. 8736551 10.1016/s0969-2126(96)00058-5

[26] Klimstra WB, Nangle EM, Smith MS, Yurochko AD, Ryman KD. 2003. DC-SIGN and L-SIGN can act as attachment receptors for alphaviruses and distinguish between mosquito cell- and mammalian cell-derived viruses. J Virol. 77:12022–12032. 14581539 10.1128/JVI.77.22.12022-12032.2003 PMC254289

[27] Shabman RS, Rogers KM, Heise MT. 2008. Ross river virus envelope glycans contribute to type I interferon production in myeloid dendritic cells. J Virol. 82:12374–12383. 18922878 10.1128/JVI.00985-08 PMC2593332

[28] Zhu D, et al 2018. Pushing the resolution limit by correcting the Ewald sphere effect in single-particle cryo-EM reconstructions. Nat Commun. 9:1552. 29674632 10.1038/s41467-018-04051-9 PMC5908801

[29] Raju S, et al 2023. A chikungunya virus-like particle vaccine induces broadly neutralizing and protective antibodies against alphaviruses in humans. Sci Transl Med. 15:eade8273. 37196061 10.1126/scitranslmed.ade8273 PMC10562830

[30] Dharmavaram S, She SB, Lázaro G, Hagan MF, Bruinsma R. 2019. Gaussian curvature and the budding kinetics of enveloped viruses. PLoS Comput Biol. 15:e1006602. 31433804 10.1371/journal.pcbi.1006602 PMC6736314

[31] Therkelsen MD, et al 2018. Flaviviruses have imperfect icosahedral symmetry. Proc Natl Acad Sci U S A. 115:11608–11612. 30348794 10.1073/pnas.1809304115 PMC6233072

[32] Carlson L-A, et al 2008. Three-dimensional analysis of budding sites and released virus suggests a revised model for HIV-1 morphogenesis. Cell Host Microbe. 4:592–599. 19064259 10.1016/j.chom.2008.10.013 PMC3454483

[33] Briggs JAG, et al 2009. Structure and assembly of immature HIV. Proc Natl Acad Sci U S A. 106:11090–11095. 19549863 10.1073/pnas.0903535106 PMC2700151

[34] Williamson LE, et al 2021. Therapeutic alphavirus cross-reactive E1 human antibodies inhibit viral egress. Cell. 184:4430–4446.e22. 34416147 10.1016/j.cell.2021.07.033 PMC8418820

[35] Kim AS, et al 2021. Pan-protective anti-alphavirus human antibodies target a conserved E1 protein epitope. Cell. 184:4414–4429.e19. 34416146 10.1016/j.cell.2021.07.006 PMC8382027

[36] Plevka P, et al 2011. Maturation of flaviviruses starts from one or more icosahedrally independent nucleation centres. EMBO Rep. 12:602–606. 21566648 10.1038/embor.2011.75 PMC3128282

[37] Kim KH, Strauss EG, Strauss JH. 2000. Adaptive mutations in Sindbis virus E2 and Ross River virus E1 that allow efficient budding of chimeric viruses. J Virol. 74:2663–2670. 10684281 10.1128/jvi.74.6.2663-2670.2000 PMC111755

[38] Strauss EG, Lenches EM, Strauss JH. 2002. Molecular genetic evidence that the hydrophobic anchors of glycoproteins E2 and E1 interact during assembly of alphaviruses. J Virol. 76:10188–10194. 12239293 10.1128/JVI.76.20.10188-10194.2002 PMC136572

[39] Sjöberg M, Garoff H. 2003. Interactions between the transmembrane segments of the alphavirus E1 and E2 proteins play a role in virus budding and fusion. J Virol. 77:3441–3450. 12610119 10.1128/JVI.77.6.3441-3450.2003 PMC149539

[40] Jose J, et al 2012. Interactions of the cytoplasmic domain of Sindbis virus E2 with nucleocapsid cores promote alphavirus budding. J Virol. 86:2585–2599. 22190727 10.1128/JVI.05860-11 PMC3302261

[41] Pulkkinen LIA, et al 2022. Molecular organisation of tick-borne encephalitis virus. Viruses. 14:792. 35458522 10.3390/v14040792 PMC9027435

[42] DiNunno NM, et al 2020. Identification of a pocket factor that is critical to Zika virus assembly. Nat Commun. 11:4953. 33009400 10.1038/s41467-020-18747-4 PMC7532219

[43] Renner M, et al 2021. Flavivirus maturation leads to the formation of an occupied lipid pocket in the surface glycoproteins. Nat Commun. 12:1238. 33623019 10.1038/s41467-021-21505-9 PMC7902656

[44] Españo E, et al 2019. Lipophilic statins inhibit Zika virus production in Vero cells. Sci Rep. 9:11461. 31391514 10.1038/s41598-019-47956-1 PMC6685969

[45] Zheng SQ, et al 2017. MotionCor2: anisotropic correction of beam-induced motion for improved cryo-electron microscopy. Nat Methods. 14:331–332. 28250466 10.1038/nmeth.4193 PMC5494038

[46] Chen M, et al 2019. A complete data processing workflow for cryo-ET and subtomogram averaging. Nat Methods. 16:1161–1168. 31611690 10.1038/s41592-019-0591-8 PMC6858567

[47] Fong RH, et al 2014. Exposure of epitope residues on the outer face of the chikungunya virus envelope trimer determines antibody neutralizing efficacy. J Virol. 88:14364–14379. 25275138 10.1128/JVI.01943-14 PMC4249124

[48] Rohou A, Grigorieff N. 2015. CTFFIND4: fast and accurate defocus estimation from electron micrographs. J Struct Biol. 192:216–221. 26278980 10.1016/j.jsb.2015.08.008 PMC6760662

[49] Henderson R, et al 2012. Outcome of the first electron microscopy validation task force meeting. Structure. 20:205–214. 22325770 10.1016/j.str.2011.12.014 PMC3328769

[50] Kimanius D, Forsberg BO, Scheres SH, Lindahl E. 2016. Accelerated cryo-EM structure determination with parallelisation using GPUs in RELION-2. Elife. 5:e18722. 27845625 10.7554/eLife.18722 PMC5310839

[51] Pintilie G, Chiu W. 2012. Comparison of Segger and other methods for segmentation and rigid-body docking of molecular components in Cryo-EM density maps. Biopolymers. 97:742–760. 22696409 10.1002/bip.22074 PMC3402182

[52] Croll TI . 2018. ISOLDE: a physically realistic environment for model building into low-resolution electron-density maps. Acta Crystallogr D Struct Biol. 74:519–530. 29872003 10.1107/S2059798318002425 PMC6096486

[53] Trabuco LG, Villa E, Mitra K, Frank J, Schulten K. 2008. Flexible fitting of atomic structures into electron microscopy maps using molecular dynamics. Structure. 16:673–683. 18462672 10.1016/j.str.2008.03.005 PMC2430731

[54] Liebschner D, et al 2019. Macromolecular structure determination using X-rays, neutrons and electrons: recent developments in *Phenix*. Acta Crystallogr D Struct Biol. 75:861–877. 31588918 10.1107/S2059798319011471 PMC6778852

[55] Kucukelbir A, Sigworth FJ, Tagare HD. 2014. Quantifying the local resolution of cryo-EM density maps. Nat Methods. 11:63–65. 24213166 10.1038/nmeth.2727 PMC3903095

[56] Pettersen EF, et al 2004. UCSF chimera—a visualization system for exploratory research and analysis. J Comput Chem. 25:1605–1612. 15264254 10.1002/jcc.20084

[57] Auguste AJ, et al 2015. Evolutionary and ecological characterization of Mayaro virus strains isolated during an outbreak, Venezuela, 2010. Emerg Infect Dis. 21:1742–1750. 26401714 10.3201/eid2110.141660 PMC4593426

