# Supplementary material for: Cryogenic electron microscopy and tomography reveal imperfect icosahedral symmetry in alphaviruses
Source: PNAS Nexus. 2024 Mar 7;3(3):pgae102. doi: 10.1093/pnasnexus/pgae102 (PMC10959069; doi:10.1093/pnasnexus/pgae102)
Supplement: pgae102_Supplementary_Data [file pgae102_supplementary_data.zip › PNASNEXUS-PNASNEXUS-2023-00830R-s04.docx]

**Table S1. Parameters for data collection and refinement statistics of CHIKV and MAYV datasets.**

| Dataset | **Single particle data** | | **Single particle data** | **Tomography**  **data** |
| --- | --- | --- | --- | --- |
| Specimen | **CHIKV** | | **MAYV** | **CHIKV-infected cell** |
| Access code |  | |  |  |
|  |  | |  |  |
| EMDB | **EMD-28979** | **EMD-41631** | **EMD-41637** | **EMD-41096** |
| PDB | **8fcg** |  |  |  |
| **Data collection and processing** |  | |  |  |
| Microscope | **Titan Krios** | | **JEM-3200FSC** | **Titan Krios** |
| Magnification | **106,000X** | | **30,000X** | **53,000X** |
| Voltage (kV) | **300** | | **300** | **300** |
| Energy filter slit width (eV) | **20** | | **20** | **20** |
| Detector | **K2 Summit** | | **K2 Summit** | **K2 Summit** |
| Defocus range (μm) | **-1 to -2.4** | | **-1.2 to -3.5** | **-3 to -5.5** |
| Pixel size (Å) | **1.34** | | **1.28** | **2.72** |
| Total electron exposure (e^-^/ Å^2^) | **47.25** | | **35** | **120** |
| Symmetry imposed | *C*_1_ | *C*_1_ | *C*_1_ | *C*_5_ |
| Map resolution (Å)  @ FSC 0.143 | **3.09** | **6.7** | **9.5** | **7.2** |
| **Refinement and model validation** |  | |  |  |
| Initial model used | **6nk5** |  |  |  |
| MolProbity score | **1.85** |  |  |  |
| Clash score | **8.18** |  |  |  |
| Rotamers outliers (%) | **0.28** |  |  |  |
| **Ramachandran plot** |  |  |  |  |
| Favored (%) | **93.94** |  |  |  |
| Allowed (%) | **5.91** |  |  |  |
| Outliers (%) | **0.15** |  |  |  |
| **R.M.S. Deviations** |  |  |  |  |
| Bond lengths (Å) | **0.007** |  |  |  |
| Bond angles | **0.702** |  |  |  |
