# Supplementary material for: Cryogenic electron microscopy and tomography reveal imperfect icosahedral symmetry in alphaviruses
Source: PNAS Nexus. 2024 Mar 7;3(3):pgae102. doi: 10.1093/pnasnexus/pgae102 (PMC10959069; doi:10.1093/pnasnexus/pgae102)
Supplement: pgae102_Supplementary_Data [file pgae102_supplementary_data.zip › PNASNEXUS-PNASNEXUS-2023-00830R-s05.docx]

**
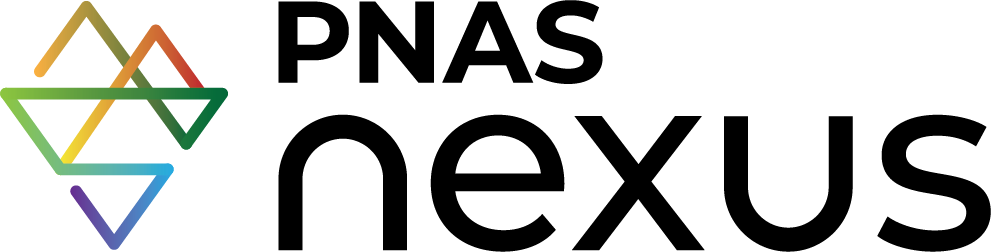
**

**Supplementary Information for**

**Cryogenic Electron Microscopy and Tomography Reveal Imperfect Icosahedral Symmetry in Alphaviruses**

David Chmielewski^a,†^, Guan-Chin Su^b,†^, Jason T. Kaelber^c^, Grigore D. Pintilie^b^, Muyuan Chen^d^, Jing Jin^e,f*^, Albert J. Auguste^g,h*^, Wah Chiu^a,b,d*^

Affiliations:

^a^Biophysics Graduate Program, Stanford University, Stanford, CA 94305, USA

^b^Department of Bioengineering and Department of Microbiology and Immunology, Stanford University, Stanford, CA 94305, USA

^c^Institute for Quantitative Biomedicine, Rutgers, the State University of New Jersey, Piscataway, NJ 08854, USA

^d^Division of CryoEM and Bioimaging, SSRL, SLAC National Accelerator Laboratory, Stanford University, Menlo Park, CA 94025, USA

^e^Vitalant Research Institute, San Francisco, CA 94118, USA

^f^Department of Laboratory Medicine, University of California, San Francisco, San Francisco, CA 94143, USA

^g^Department of Entomology, College of Agriculture and Life Sciences, Fralin Life Science Institute, Virginia Polytechnic Institute and State University, Blacksburg, VA 24061, USA

^h^Center for Emerging, Zoonotic, and Arthropod-borne Pathogens, Virginia Polytechnic Institute and State University, Blacksburg, VA 24061, USA

*Correspondence to:

Jing Jin, Email:jjin@vitalant.org

Albert Auguste, Email: jauguste@vt.edu

Wah Chiu, Email: [wahc@stanford.edu](mailto:wahc@stanford.edu)

**This file includes:**

Fig. S1 to S10

**Other supplementary materials for this manuscript include the following:**

Table S1

Movies S1 to S3

**Supplementary figures**


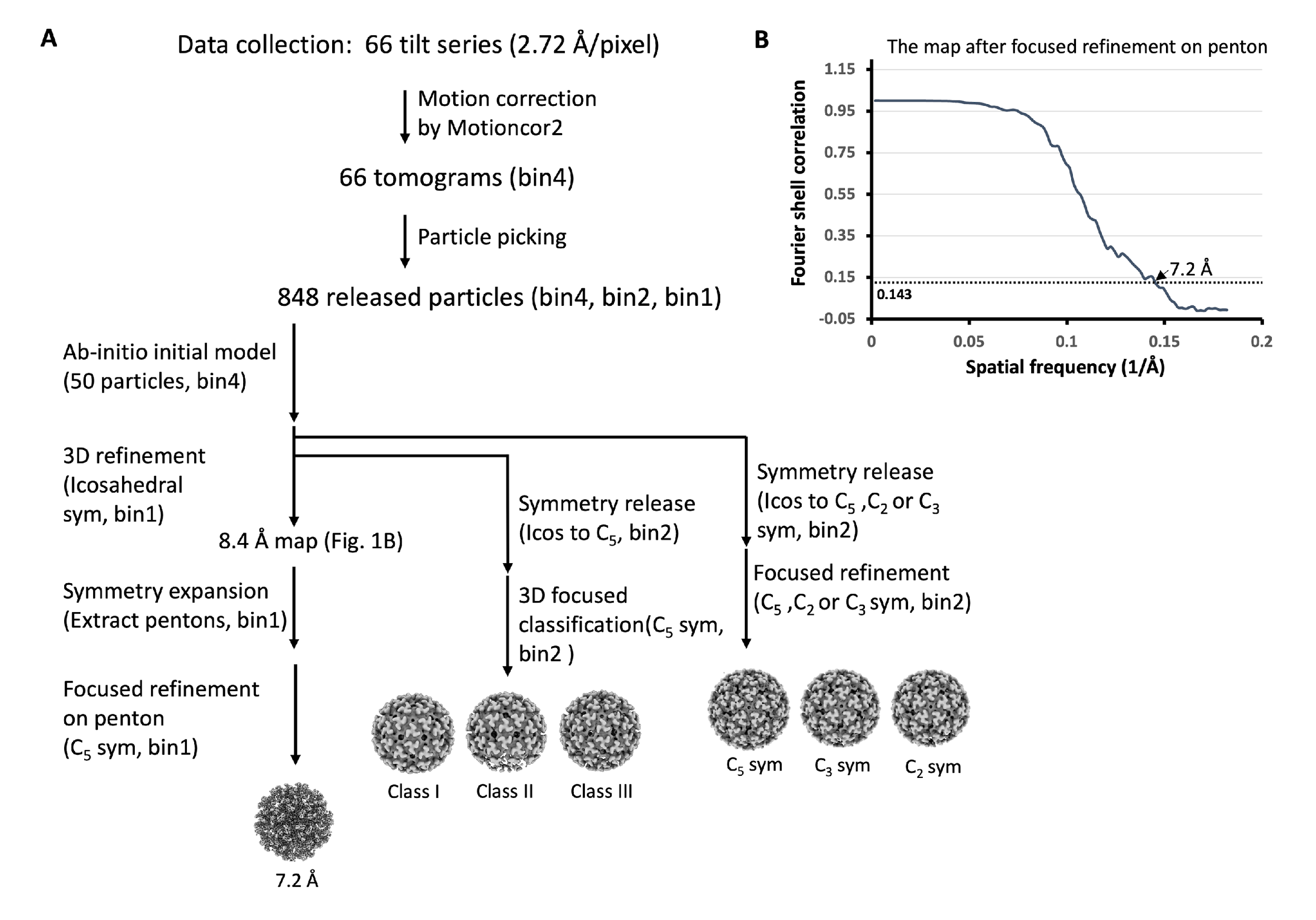


**Fig. S1. Cryo-ET data processing workflow of Chikungunya virus.** (A) Cryo-ET and data processing workflow for release virions. (B) Gold standard Fourier shell correlation (FSC) plots of CHIKV pentamer structure with focused refinement.


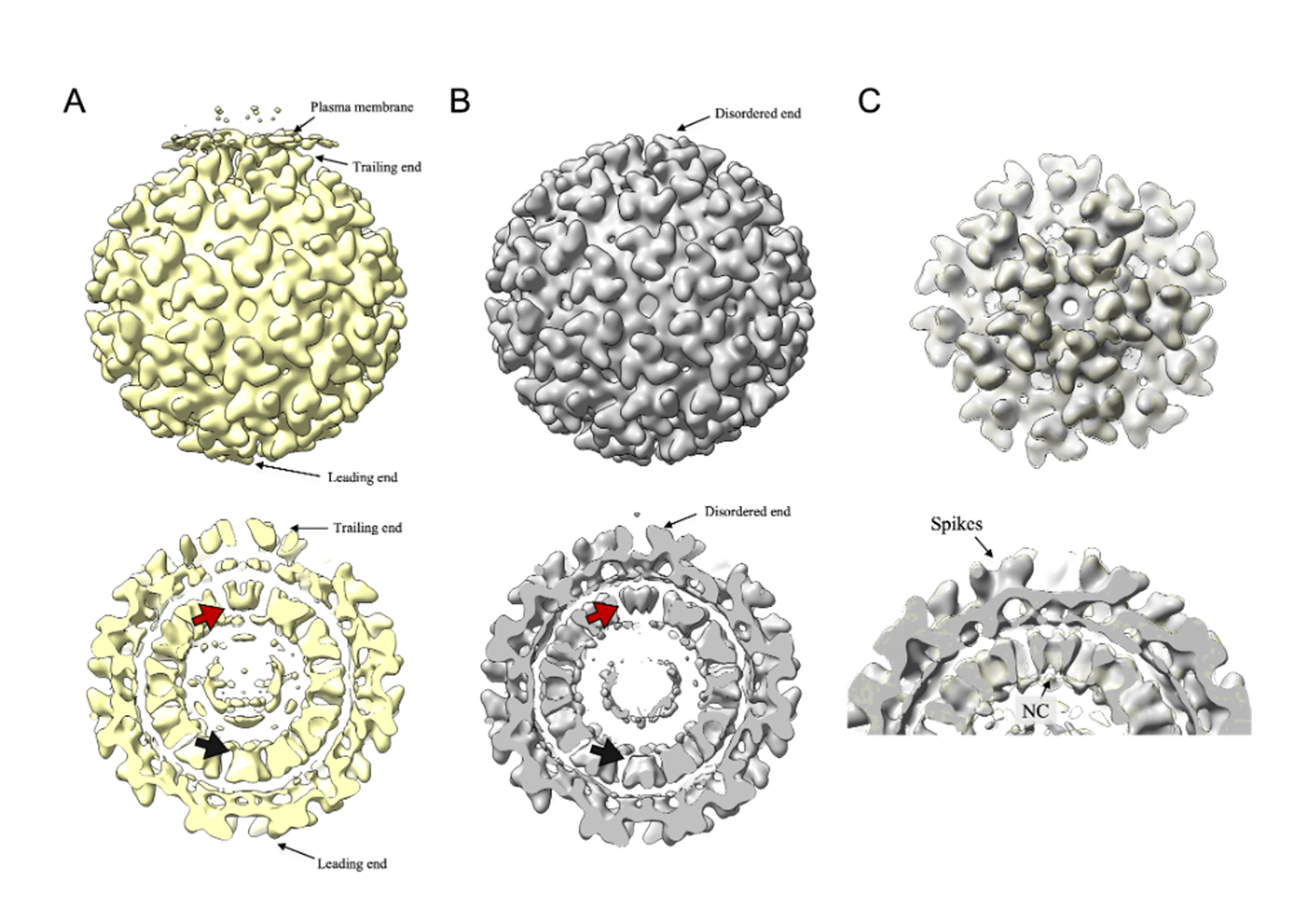


**Fig. S2. Comparison of late-stage CHIKV budding particle to released virion.** (A) Subtomogram average density map (top) of late stage CHIKV budding intermediate still connected to the plasma membrane with half-cut representation (bottom). Leading end and trailing end (connected to membrane) labeled. Weak penton density within the NC layer (red) and normal penton density (black) labeled with arrows. (B) Subtomogram average of released CHIKV virion (Fig. 1D, class III) with half-cut representation (bottom). Weak density NC penton (red) and normal NC penton density (black) labeled with arrows. (C) Alignment of density maps in (A) & (B) with top view of the virion surface spikes (top) and side view with half-cut representation (bottom) reveals close overall agreement between “trailing end” of map in (A) and “disordered end” of map in (B).


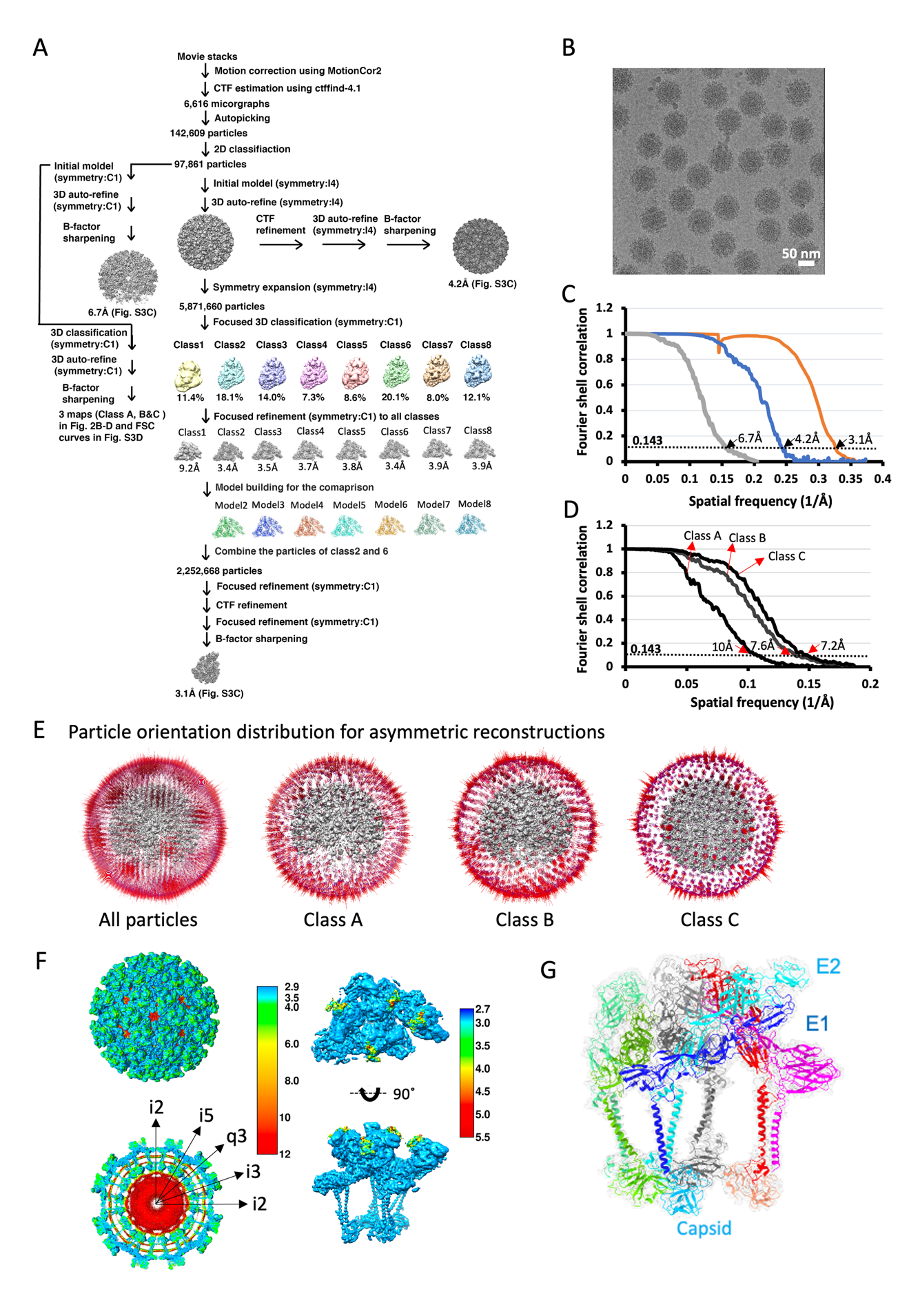


**Fig. S3. Cryo-EM of purified Chikungunya virus.** (A) Single particle cryo-EM data processing workflow for CHIKV. (B) Representative micrograph of purified CHIKV particles. (C) FSC curves for the virion map with asymmetric reconstruction (gray), the virion map with icosahedral reconstruction (blue) and the ASU map after focused refinement with *C*_1_ symmetry of Class 2+Class 6 ASUs (orange). (D) FSC curves for the virion maps with asymmetric reconstruction of class A, class B and class C of 3D classification with *C*_1_ symmetry (from left to right). (E) Euler angle distribution of the particles used in asymmetric reconstructions of whole virion particles. (F) The virion map with icosahedral reconstruction (left), with extracted central section (below) and ASU map after focused refinement (right) colored by estimated local resolution. (G) Atomic model was built using the ASU map at 3.09 Å. E1, E2 and capsid protein used the same coloring scheme in Fig. 4B(II).


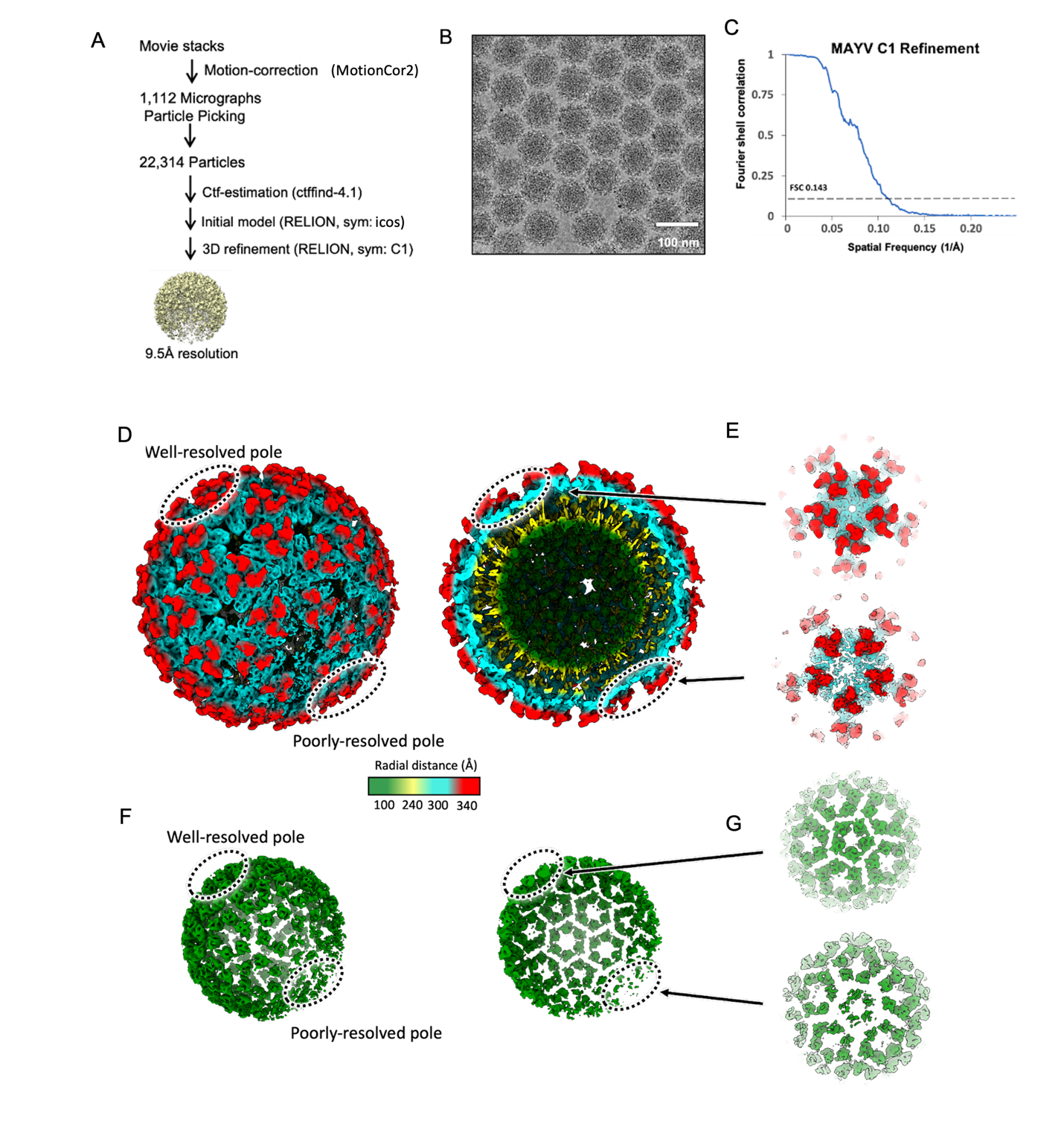


**Fig. S4. Asymmetric cryo-EM reconstruction of purified Mayaro virus reveals weak icosahedral features at one pole.** (A) Single particle cryo-EM data processing workflow for MAYV. (B) Representative micrograph of purified MAYV particles. (C) FSC curves for MAYV *C*_1_ refinement. (D) Radially-colored surface of the asymmetric reconstruction of the MAYV particle (left) with half-cut view to reveal particle interior (right). (E) Zoom-in view of the “well-resolved pole” (top) and “poorly-resolved pole” (bottom) displayed at sufficient density threshold to view features of trimeric spikes. (F) Computationally-extracted density of the nucleocapsid from the MAYV asymmetric reconstruction in Fig. S4D (left) with half-cut representation (right). (G) Zoom-in views of the NC focused on the Cp penton assembly at both the “well-resolved pole” (top) and “poorly-resolved pole” (bottom).


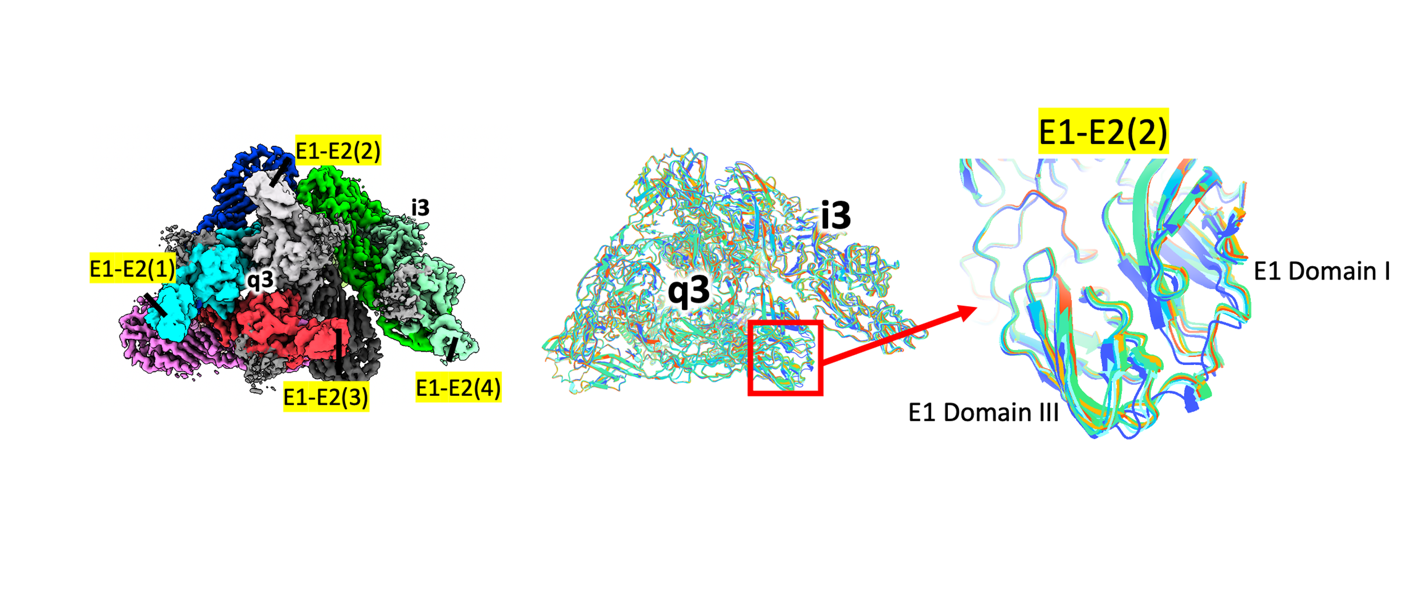
**Fig. S5. A conformational difference among CHIKV ASU models.** After focused classification, the particles in each class displayed in percentage of total particles were grouped and refined. Models were built into the ASU maps below 4 Å (class 2-8) (Fig. S3A). The 7 models built from ASU maps of class 2-8 were aligned for comparison of the glycoprotein conformations (Fig. S3A). Significant conformational difference was found in the q3 E1-E2 dimer 2 of model 3 colored by blue compared to other models and attributed to a distinct conformation of the linker region between E1 domain I and III within E1-E2 dimer 2 of model 3 (blue).


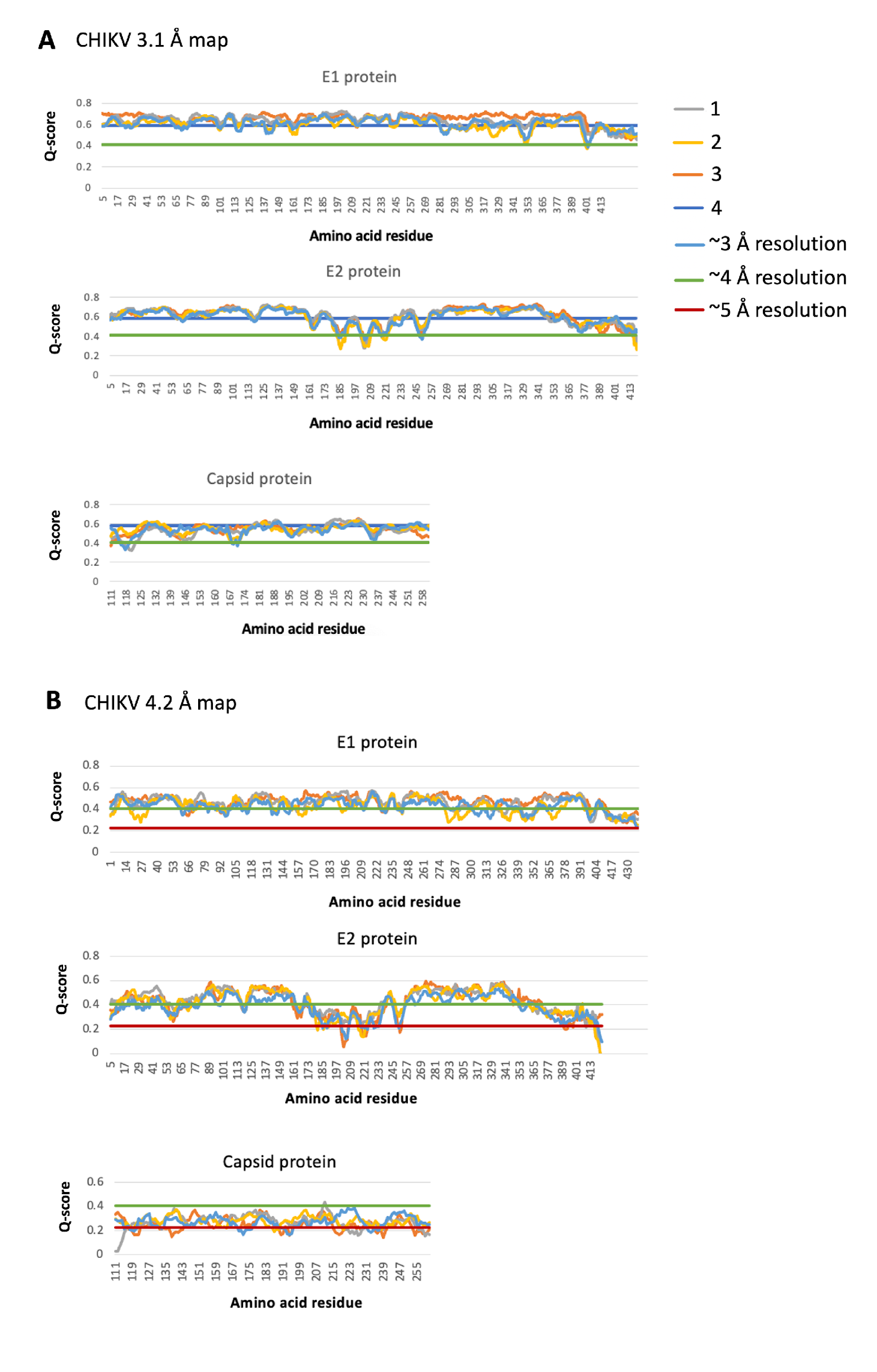


**Fig. S6. Q-scores analysis of cryo-EM maps.** Q-scores analysis for E1, E2 and capsid protein in (A) CHIKV 3.1 Å map after focused classification and focused refinement with *C*_1_ symmetry, (B) CHIKV 4.2 Å map with icosahedral refinement.


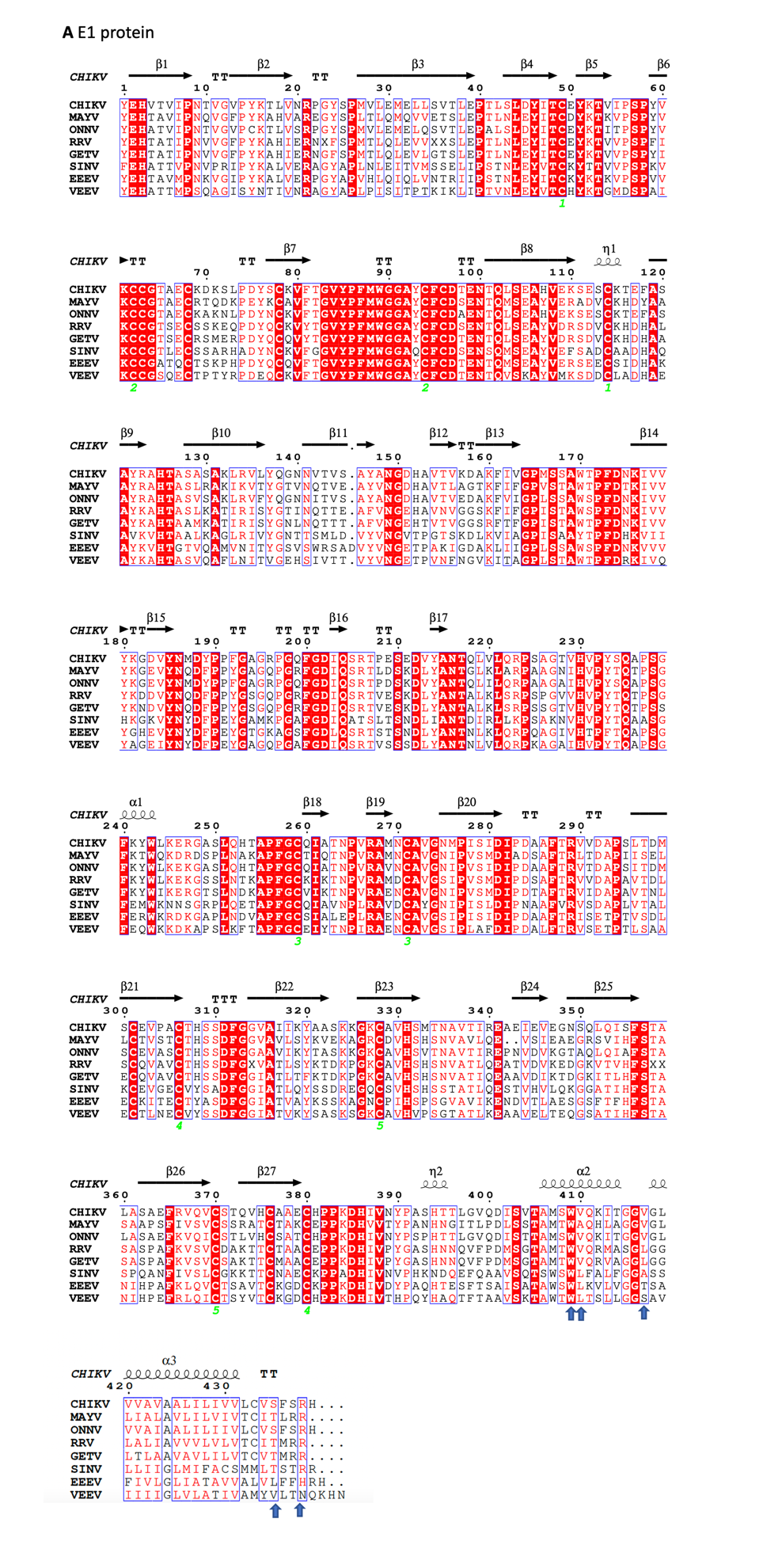

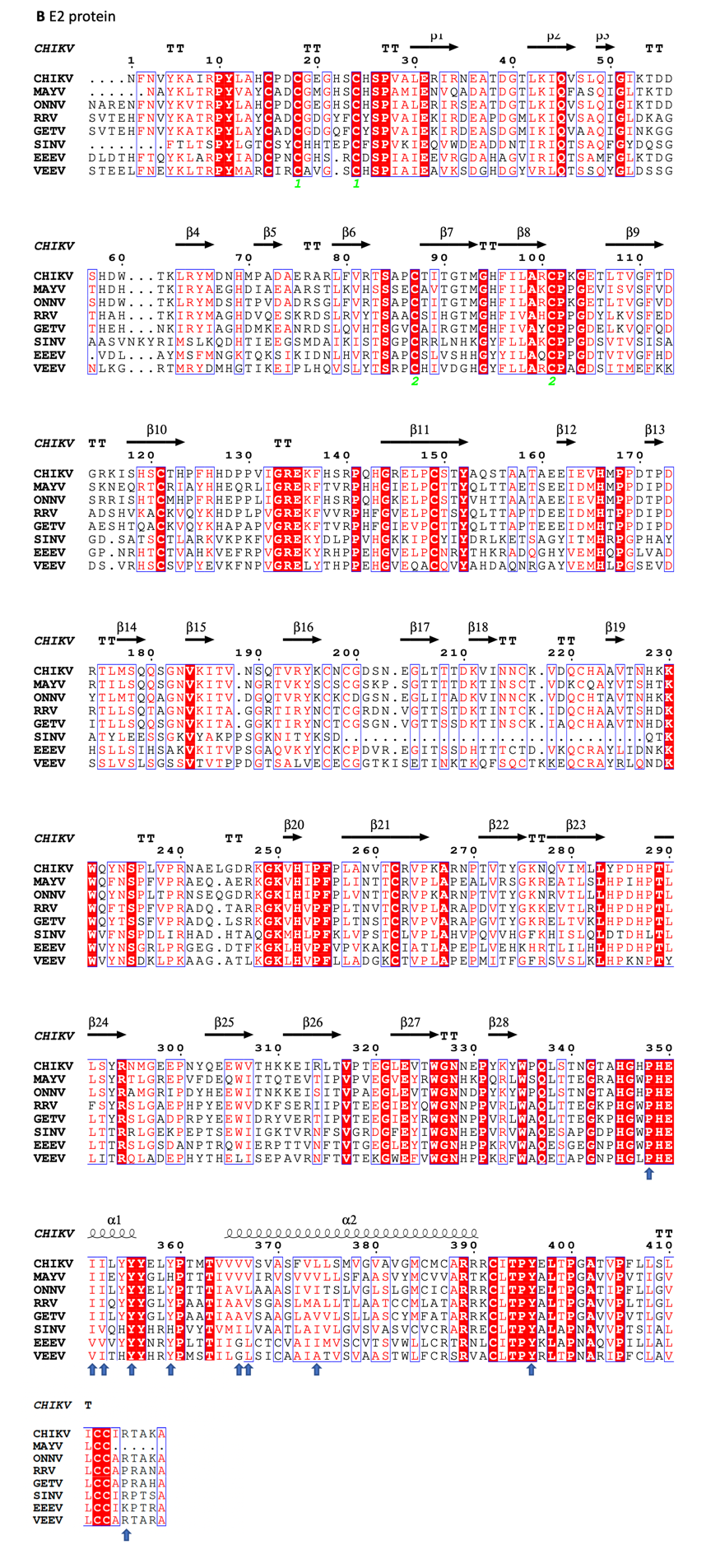


**Fig. S7. Sequence alignments of the E1 and E2 proteins among different alphaviruses.** In (A) E1 protein and (B) E2 protein, conserved residues are boxed and coloured red. Completely conserved residues are shown in white on a red background. Blue arrows underneath the alignments indicate conserved residues involved in the interactions related to Fig. 5 and Fig. 6.


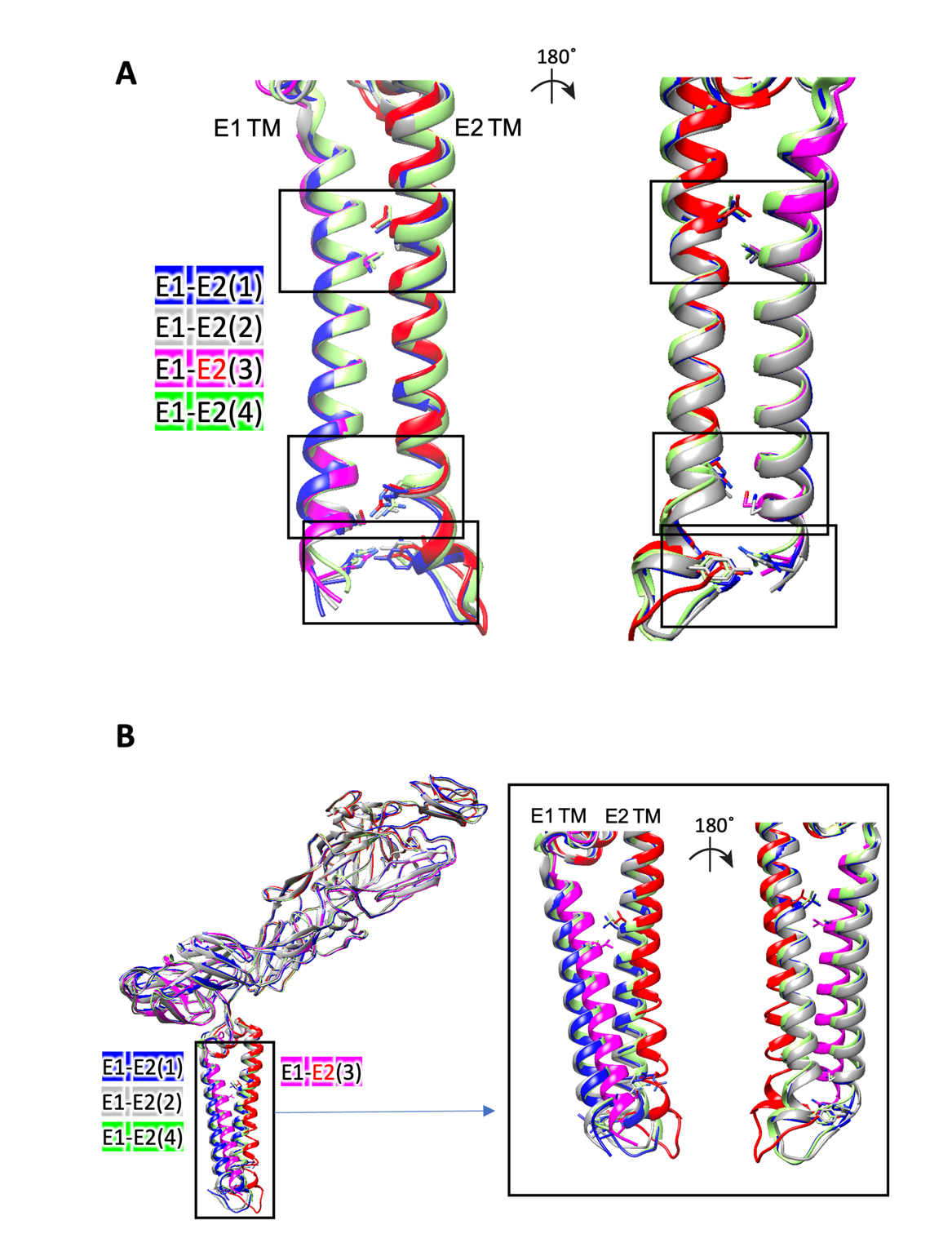


**Fig. S8**. **The interaction and conformation among four E1-E2 heterodimers in CHIKV ASU.** (A) The alignment of four E1-E2 TM helices from CHIKV ASU. E1-E2 TM helix structure among four E1-E2 heterodimers is similar. The interactions identified in figure 5 are marked in the black squares. (B) A conformational difference among four E1-E2 heterodimers in ASU. The conformation of E1_(3)_-E2_(3)_ TM helices is different to others.


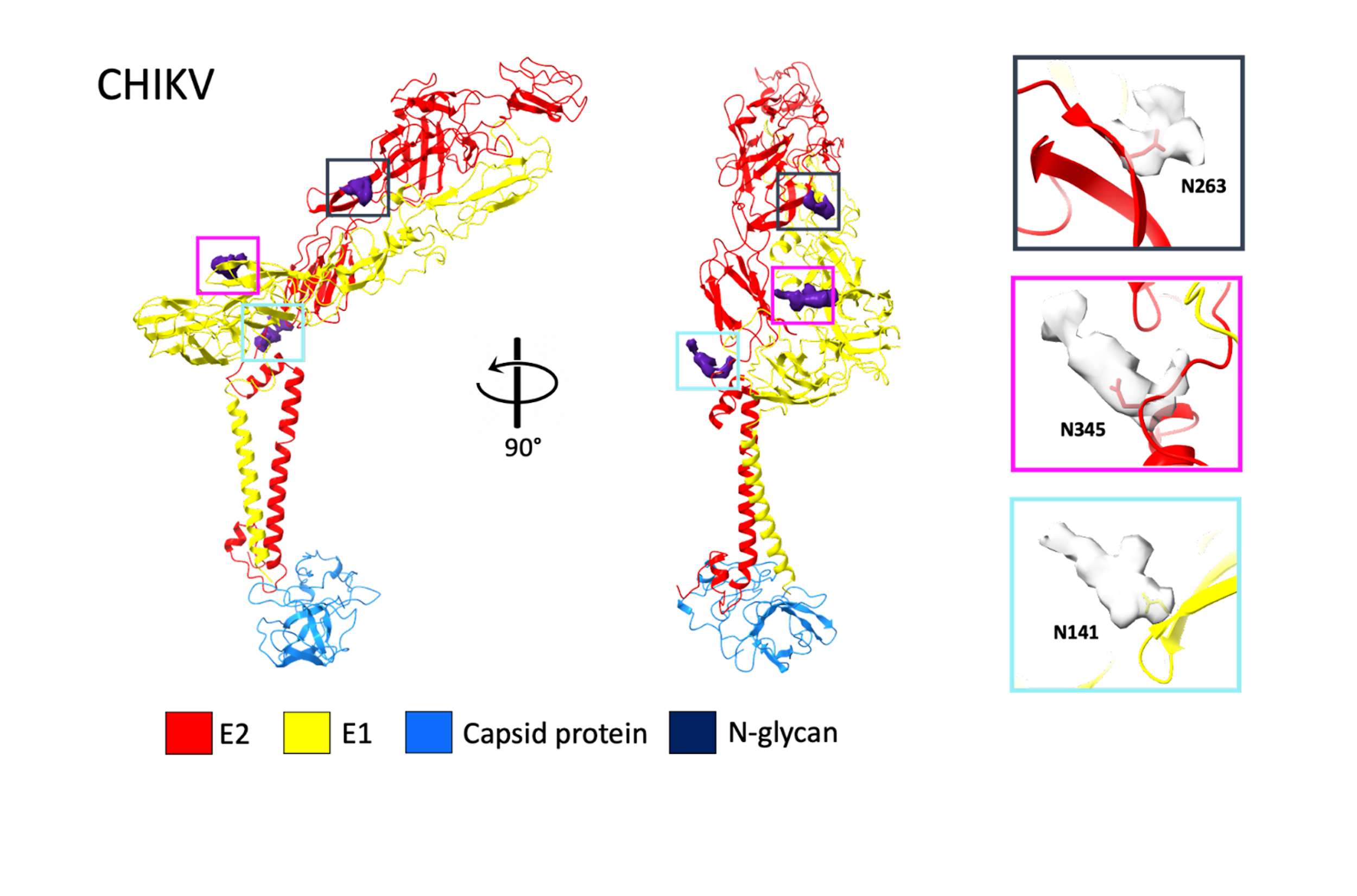


**Fig. S9. Glycan density in CHIKV map.** CHIKV E1-E2-Cp subunit models with cryo-EM density (purple) from the map displayed at the predicted sites of N-linked glycosylations. Zoom-in views of glycosylation sites (colored boxes) show the extra density originating from the Asn residues, unaccounted for by the atomic model and attributed to glycans.


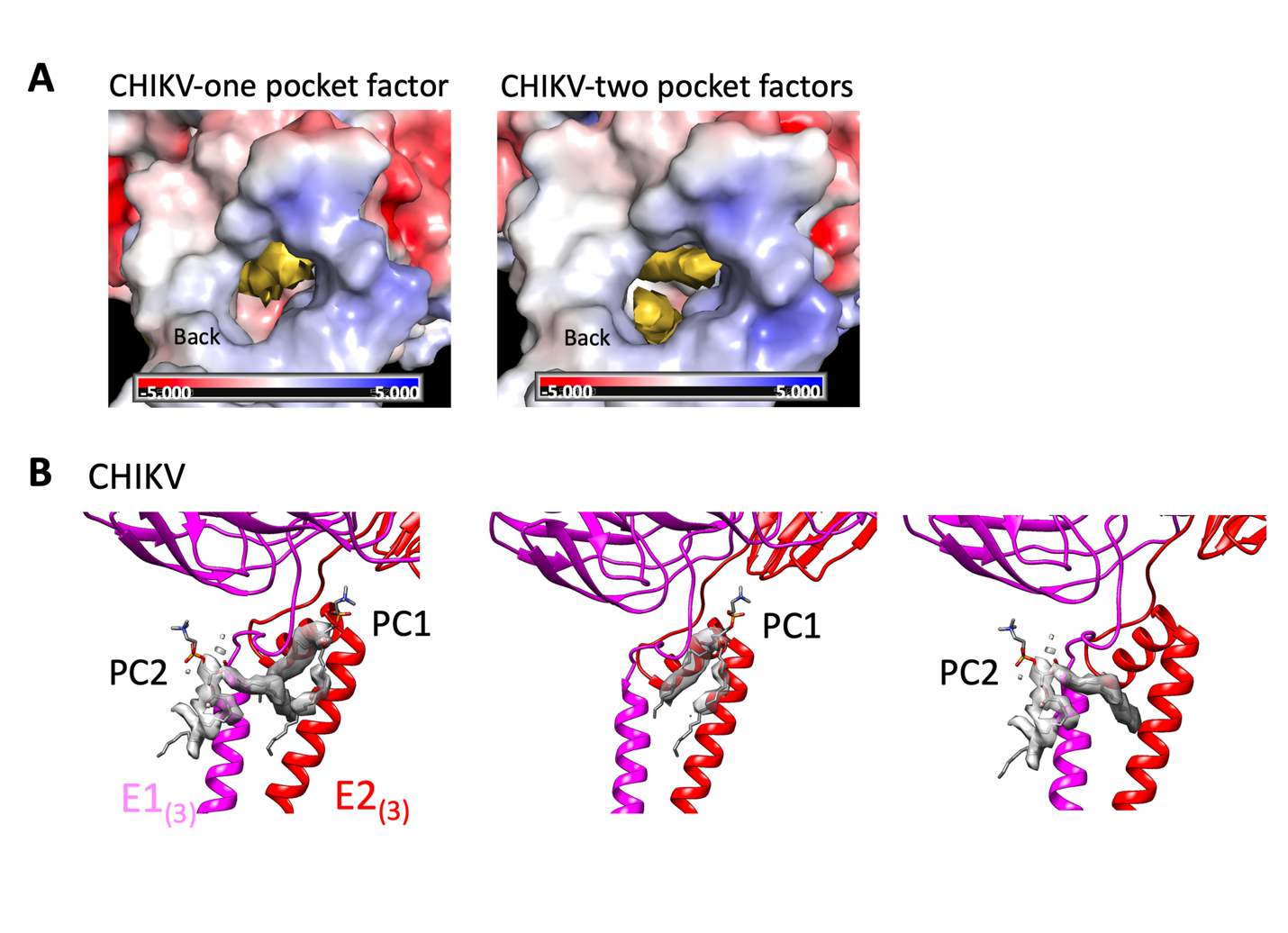


**Fig. S10. Pocket factor in the hydrophobic pocket of CHIKV.** (A) CHIKV hydrophobic pocket surface colored by electrostatic potential and pocket factor colored with yellow. (B) The fitting of two phospholipid molecules (Phosphatidylcholine (PC)) into the two extra densities in the E1-E2 dimer 3 in CHIKV ASU.

**Supplementary table**

**Table S1.** Parameters for data collection and refinement statistics of CHIKV and MAYV datasets.

**Supplementary movies**

**Movie S1.** Tomogram of released virion in the periphery region

**Movie S2.** Morph between subtomogram average class I and class III (top view).

**Movie S3.** Morph between subtomogram average class I and class III (side view).
