# Supplementary figures and images for: Cryogenic electron microscopy and tomography reveal imperfect icosahedral symmetry in alphaviruses
Source: PNAS Nexus. 2024 Mar 7;3(3):pgae102. doi: 10.1093/pnasnexus/pgae102 (PMC10959069; doi:10.1093/pnasnexus/pgae102)

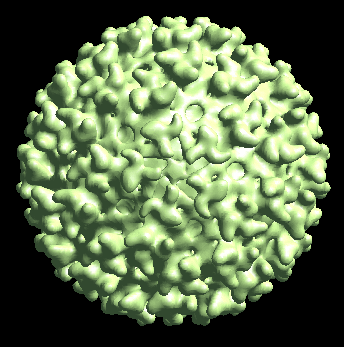

Supplement: pgae102_Supplementary_Data [file pgae102_supplementary_data.zip › PNASNEXUS-PNASNEXUS-2023-00830R-s02.gif]

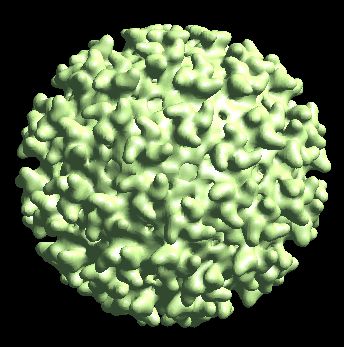

Supplement: pgae102_Supplementary_Data [file pgae102_supplementary_data.zip › PNASNEXUS-PNASNEXUS-2023-00830R-s03.gif]
